# Complications after knee derotational osteotomies in patients with anterior knee pain and/or patellofemoral instability: a systematic review with meta-analysis

**DOI:** 10.1530/EOR-2024-0036

**Published:** 2025-01-03

**Authors:** Inês Figueiredo, Cristina Valente, Ricardo Ribeiro, Bárbara Ferreira, Eluana Gomes, Diego Delgado, Mikel Sánchez, Renato Andrade, João Espregueira-Mendes

**Affiliations:** ^1^ School of Medicine, Minho University, Braga, Portugal; ^2^ Clínica Espregueira-FIFA Medical Centre of Excellence, Porto, Portugal; ^3^ Dom Henrique Research Centre, Porto, Portugal; ^4^ Advanced Biological Therapy Unit, Hospital Vithas Vitoria, Vitoria-Gasteiz, Spain; ^5^ Arthroscopic Surgery Unit, Hospital Vithas Vitoria, Vitoria-Gasteiz, Spain; ^6^ Porto Biomechanics Laboratory (LABIOMEP), Faculty of Sports, University of Porto, Porto, Portugal; ^7^ ICVS/3B’s–PT Government Associate Laboratory, Braga/Guimarães, Portugal; ^8^ 3B’s Research Group-Biomaterials, Biodegradables and Biomimetics, University of Minho, Headquarters of the European Institute of Excellence on Tissue Engineering and Regenerative Medicine, Barco, Guimarães, Portugal

**Keywords:** anterior knee pain, derotational, femur, osteotomy, patellofemoral instability, tibia

## Abstract

**Purpose:**

- Investigate intra- and post-operative complications and revisions following distal femoral and/or high tibial derotational osteotomies to correct rotational malalignments of the lower limb in patients with anterior knee pain (AKP) and/or patellofemoral instability (PFI).

**Methods:**

- A literature search was conducted on PubMed, EMBASE and Web of Science (until 30 September 2023), including studies reporting complications, reinterventions and revisions following knee derotational osteotomies. Incidence rates were collected for each level of derotational osteotomy (distal femur, high tibia or double-level). A meta-analysis using the Freeman–Tukey double arcsine transformation was conducted to estimate the pooled proportions with their 95% confidence intervals (CIs).

**Results:**

- Twenty-one studies involving 564 osteotomies (*n* = 484) were included, with a mean follow-up of 45.6 ± 15.7 months. The overall complication proportion was 7.5% (95% CI: 3.9–11.8%). Postoperative residual AKP was seen in a pooled proportion of 7.6% (95% CI: 0.7–18.8%), and persistent PFI was not common (0.1%; 95% CI: 0.0–1.7%). Intraoperative complications occurred in a pooled proportion of 3.8% (95% CI: 2.4–6.0%), with peroneal nerve injury being the most common (1.3%) after derotational high tibial osteotomy. Reintervention was needed in a pooled proportion of 13.0% (95% CI: 2.9–27.2%), primarily for hardware removal (*n* = 158; 28.3%). There was a pooled proportion of knees requiring revision procedures of 12.3% (95% CI: 2.6–26.1%).

**Conclusions:**

- Distal femur and high tibial derotational osteotomies exhibit a considerable incidence of intra- and post-operative complications. Peroneal nerve injury, although infrequent, is a significant complication, underscoring the importance of implementing intraoperative preventive measures during derotational high tibial osteotomy.

## Introduction

Patellofemoral joint stability is ensured by a complex interplay of a variety of inter-dependent static and dynamic risk factors (1, 2). Several pathoanatomical factors contribute to patellofemoral instability (PFI) and anterior knee pain (AKP), including genu valgum or varum, deficient or ruptured medial patellofemoral ligament (MPFL), patella alta, trochlear dysplasia, increased tibial tuberosity trochlear grove distance and lower limb malalignments among other risk factors (2, 3, 4, 5, 6, 7, 8, 9, 10).

Lower limb torsional abnormalities can be due to excessive femoral anteversion (11) or excessive tibial torsion (12). The rotation of the lower limb adds a lateral vector to the patella, counteracted by soft tissue (medial and lateral patellofemoral soft tissue restrainers) and by the trochlea anatomy, resulting in abnormal patellar tension placed on the capsule, ligaments, synovium or subchondral bone and, consequently, leading to AKP and PFI (2, 13, 14, 15).

Knee derotational osteotomy was introduced to neutralise the lateralised patellar force vector in patients with lower limb torsional abnormalities and, consequently, to reduce its biomechanical consequences (16). During a knee derotational osteotomy, a cut is made, and then, both fragments are rotated to achieve the desired correction (13). Derotational distal femoral osteotomy (DDFO) and/or derotational high tibial osteotomy (HTO) are performed to correct rotational malalignment, thereby helping to reduce AKP and PFI, with good to excellent patient satisfaction and improved patient-reported outcomes (17, 18).

The scientific literature on the incidence of intra- and post-operative complications after DDFO and derotational HTO in individuals with AKP and/or PFI remains poorly described and systematised. There is a previous systematic review on the topic (19), but it had a broader scope, incorporating proximal femoral and distal tibial derotational osteotomies. The description and summary on the incidence of complications and reinterventions after knee derotational osteotomies is clinically relevant and necessary to inform the orthopaedic surgeon on which complications are more frequent and which may lead to the need of a reintervention or revision surgery. Ultimately, this may help the orthopaedic community to implement preventive strategies to mitigate the risk of complications. Therefore, the aim of our systematic review was to evaluate the incidence of intra- and post-operative complications, reinterventions, revisions and conversions to total knee arthroplasty (TKA) following knee rotational osteotomy to correct lower limb rotational malalignments in patients with AKP and/or PFI.

## Methods

This systematic review was conducted following the PRISMA (Preferred Reporting Items for Systematic Reviews and Meta-Analyses) 2020 statement (19) and following the recommendations of the PRISMA PERSiST guidance (20). The protocol for this systematic review was *a priori* registered in PROSPERO under no. CRD42023401305.

### Eligibility criteria

The eligibility criteria were framed according to the Participants, Intervention, Comparison, Outcomes, and Study Design (PICOS) strategy. When eligible studies had overlapping samples, the study with the largest sample size was retained.

#### Participants

Studies comprising individuals with AKP and/or PFI treated with DDFO and/or derotational HTO were included, regardless of age, sex, race or health status. Participants with a history of prosthetic placement, traumatic injury to the knee, infection, tumour or any congenital and developmental disorders (e.g. cerebral palsy, Legg–Calvé–Perthes or dysplasia) were excluded.

#### Intervention

Studies were included if DDFO and/or derotational HTO was performed to correct lower limb malalignments in patients with AKP and/or PFI. Double-level derotational osteotomies were allowed only if performed at the knee joint (distal femur and proximal tibia). Studies describing concomitant surgical interventions, including fibular osteotomy, MPFL reconstruction, tibial tubercle transfer (TTT), lateral retinaculum release or trochleoplasty, were incorporated, except when involving associated TKA or patellar arthroplasty.

#### Comparator

A comparator group is not required, but, if available, the main intervention was compared based on the level of derotational osteotomy (distal femur vs proximal tibia).

#### Outcomes

The intra- and post-operative complications, reinterventions or conversions to TKA after DDFO and/or derotational HTO were considered as the primary outcomes.

#### Study design

All clinical trials that reported complications, reinterventions and conversions to TKA incidence, spanning from randomised controlled trials, non-randomised controlled trials, cohort clinical trials, case–control studies to case series, were included. All other types of studies (e.g. case studies, cross-sectional studies and other systematic reviews) were excluded. Due to restrains in the availability of translation resource, only studies in English language were included.

### Search strategy

A systematic and comprehensive online literature search was conducted on PubMed, EMBASE and Web of Science databases on 27 March 2023 and updated on 30 September 2023. The full search strategy for each database is reported in Supplementary Table 1 (see section on Supplementary materials given at the end of the article).

### Study selection

All studies identified were downloaded to Mendeley Reference Manager 2.84.0 for MacOS (https://www.mendeley.com/release-notes-reference-manager/v2.84.0). Subsequently, an automated process identified and eliminated duplicated studies, and then checked manually. Two authors (IF and RA) meticulously examined each title and abstract to evaluate their potential relevance for inclusion. Relevant studies underwent full-text evaluation to further determine their adherence to the predetermined eligibility criteria. All references from the included studies were screened manually to verify that no relevant studies were missing from the systematic review. Disagreements between authors on full-text analysis were discussed until consensus.

### Data collection and management

Two authors (IF and RA) organised data into predetermined tables in a Microsoft® Excel spreadsheet, incorporating the data defined *a priori* (Supplementary Text 1).

Continuous data were collected as mean and standard deviation, and categorical data were collected as number and percentage (%). In cases where only the median was provided, the mean value was estimated using the methods proposed by Hozo and coworkers (21) to calculate the weighted follow-up, and in cases where only a value range is provided, the lowest value was used. Pooled summary metrics were weighted to the sample size.

Postoperative complications were classified according to the modified Clavien–Dindo scale for complications in orthopaedic surgery (22) and then subgrouped into minor and major complications (Supplementary Text 2).

### Risk of bias

Two authors (IF and RA) judged the risk of bias of each study using the Risk of Bias Assessment tool for Non-Randomized Studies (RoBANS) (23). The RoBANS is a validated instrument, encompassing six domains of bias: (i) selection of participants; (ii) confounding variables; (iii) exposure measurement; (iv) blinding of outcome assessment; (v) incomplete outcome data; and (vi) selective outcome reporting (Supplementary Table 2). Each domain was judged as low risk of bias, high risk of bias or unclear. Disagreements were discussed until consensus.

### Data synthesis

All outcome data were summarised as overall frequency and percentage (of the total considered sample). The number of knees (rather than sample size) was used to calculate the outcome rates.

Meta-analysis was conducted using the ‘meta’, ‘metadata’ and ‘metafor’ packages with the R Studio (version 2024.04.2+764; https://dailies.rstudio.com/version/2024.04.2+764/) software. Due to the lack of homogenous control groups, a comparative meta-analysis (for relative risk) was not possible. Therefore, a meta-analysis of proportions was performed using a random-effects model (inverse variance) to pool the proportion of complications of all included studies. Proportions were transformed using a Freeman–Tukey double arcsine transformation (24) and zero events were corrected using continuity correction adding 0.5 in each cell. The pooled results of meta-analysis were expressed as proportions and 95% confidence intervals (CIs). The 95% CIs were adjusted using the Clopper–Pearson method to adjust for smaller sample sizes (25). Heterogeneity across individual studies was explored using the Cochran’s Q statistical test and *I*^2^ index (*I*^2^ values of 25, 50 and 75% represent low, moderate and high heterogeneity, respectively) (26). Risk of publication bias was assessed when meta-analysis included at least 10 studies (27) by visual inspection of the funnel plot.

The meta-analysis was conducted for complications (overall complications, postoperative complications, intraoperative complications, residual AKP and residual PFI) and subgrouped by the level of knee derotational osteotomy (derotational HTO, DDFO, double-level or mixed). The mixed derotational subgroup lumps the studies with both derotational HTO and DDFO where the number of complications for each level of osteotomy could not be determined. A separate meta-analysis was performed to calculate the pooled proportion of reinterventions and revisions.

Sensitivity analyses were conducted by rerunning the analyses (overall complications and reinterventions) whilst excluding studies with: short follow-up duration (less than 24 months); remove reinterventions where the removal hardware may have been planned and not due to other causes (three studies) (28, 29, 30); and remove one study (28) where there was a single case of double-level osteotomy mixed with cases of DDFO.

Meta-regression analyses were performed to investigate the presence of unexplained heterogeneity considering three covariates that included mean age, mean follow-up duration and level of osteotomy.

## Results

### Study selection

**Figure 1:**
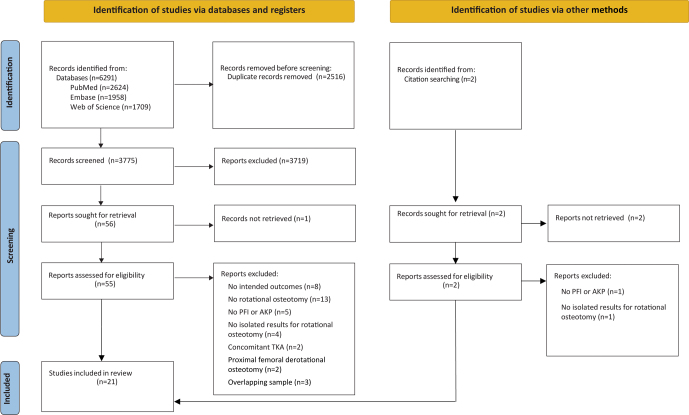
PRISMA 2020 flow diagram depicting the exclusion/inclusion process. AKP, anterior knee pain; PFI, patellofemoral instability; TKA, total knee arthroplasty.

The electronic and hand searches resulted in 6293 studies. After removing 2516 duplicates, 3777 articles were assessed based on titles and abstracts. Most of these studies were not related to knee derotational osteotomies and were thus excluded based on their abstract screening. A total of 57 studies proceeded to a full-text assessment, of which 21 studies were included (Fig. 1) (28, 29, 30, 31, 32, 33, 34, 35, 36, 37, 38, 39, 40, 41, 42, 43, 44, 45, 46, 47, 48).

### Risk of bias

**Figure 2:**
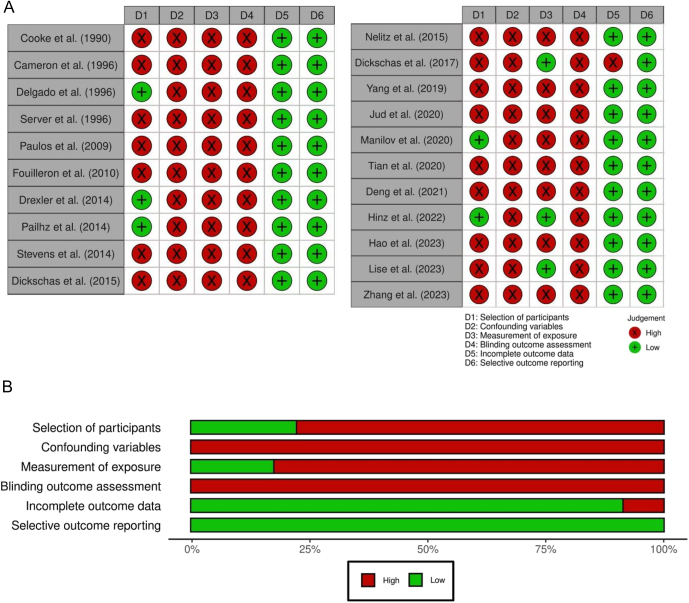
Risk of bias plots using RoBANS: (A) traffic light plot and (B) weight summary plot of all included studies (28, 29, 30, 31, 32, 33, 34, 35, 36, 37, 38, 39, 40, 41, 42, 43, 44, 45, 46, 47, 48).

All the studies were judged with a high risk of bias in at least one domain, with 15 studies (71%) (29, 30, 32, 34, 36, 37, 39, 40, 41, 42, 43, 44, 46, 47, 48) having four or more domains identified as high risk (Fig. 2). Specifically, the ‘selection of participants’ domain was judged as high risk of selection bias in 16 studies (76%), primarily due to the inclusion of individuals with both AKP and PFI within the same sample. All studies were evaluated as high risk of selection bias in the ‘confounding variables’ domain, attributable to the heterogeneity in age, sex and previous and concomitant surgeries among the patients. Almost all studies (86%) were considered as having high risk of performance bias owing to their retrospective nature. All studies were judged to be at high risk for detection bias because outcome assessors were not blinded. Conversely, only one study was identified as having a high risk of attrition bias due to missing data (32) and none was judged as high risk of reporting bias.

### Study characteristics and patients’ demographics

Included studies were published in English between 1990 and 2023, taking place mostly in China (*n* = 5) and Germany (*n* = 5). The mean follow-up duration was 45.2 ± 15.4 months. There were 484 patients (mostly females, 86%), with a total of 564 derotational knee osteotomies. The average age of the participants was 24.1 ± 4.6 years, with a BMI of 23.0 ± 2.0 kg/m^2^. Eleven studies reported the knee laterality (343 knees) (29, 30, 31, 37, 38, 42, 44, 45, 46, 47, 48), with 47% (*n* = 161) undergoing surgical procedures on the left side and 53% (*n* = 182) on the right side. Nine studies examined patients receiving treatment for PFI (28, 30, 34, 35, 40, 43, 44, 47, 48), four studies for AKP (33, 36, 38, 45) and eight studies for both AKP and PFI (29, 31, 32, 37, 39, 41, 42, 46) (Supplementary Table 3).

### Surgical intervention characteristics

There were 274 isolated DDFOs (12 studies), 253 isolated derotational HTOs (10 studies) and 37 double-level osteotomies (4 studies). One study (28) performing DDFO included only one double-level osteotomy and was thus accounted for DDFO. More than 35% (*n* ≥ 196) of the knees underwent combined MPFL reconstruction (28, 30, 31, 32, 34, 44, 46, 47, 48), 28% (*n* = 157) underwent combined lateral retinaculum procedures (lengthening or release) (28, 31, 36, 39, 41, 42, 44, 45), and 10% (*n* = 58) underwent combined TTT (28, 30, 35, 40, 41, 44). Other concomitant procedures are reported in Supplementary Table 4. Twelve studies (28, 29, 31, 34, 35, 36, 37, 39, 40, 41, 42, 45) documented previous knee surgical procedures, most commonly lateral retinaculum release (*n* ≥ 33; ≥5.9%) (29, 35, 36, 37, 41, 42, 45) and TTT (*n* ≥ 29; ≥5.1%) (29, 35, 41, 42, 45).

### Complications

**Figure 3:**
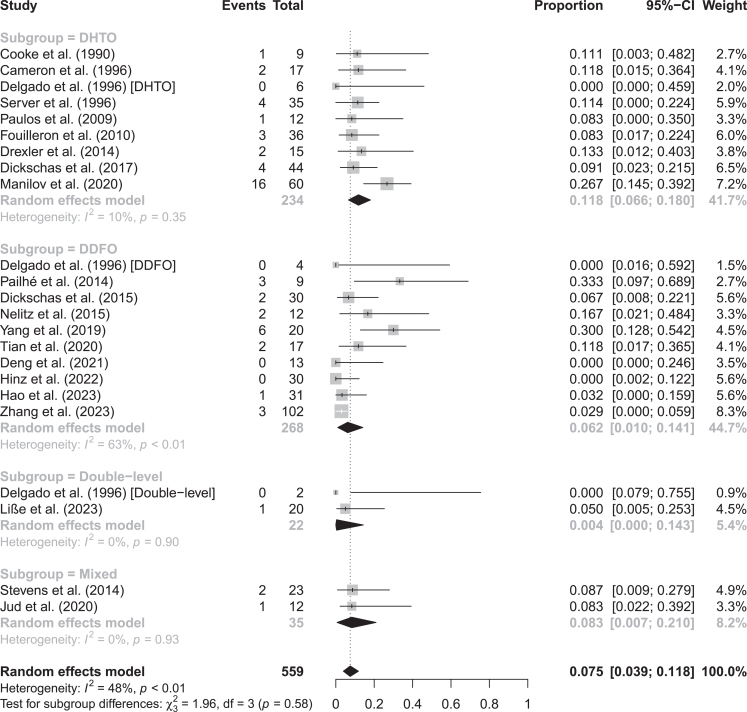
Forest plot on proportions of overall complications subgrouped by the level of osteotomy (28, 29, 30, 31, 32, 33, 34, 35, 36, 37, 38, 39, 40, 41, 42, 43, 44, 45, 46, 47, 48).

The overall proportion of complications (intra- and post-operative) following derotational knee osteotomies was 7.5% (95% CI: 3.9–11.8%, *I*^2^ = 48%). Although there was a higher proportion of overall complications for derotational HTO (Table 1), there were no significant differences between subgroups based on the level of derotational osteotomy (Fig. 3). Removing the studies with short follow-up (<24 months) did not increase the pooled proportion of complications; however, for the DDFO subgroup, it decreased to 1.5% (95% CI: 0.0–4.3%) with a notable decrease in heterogeneity (from *I*^2^ = 63 to 0%; Supplementary Table 5). Meta-regression showed that derotational HTO had an increasing effect on the proportion of complications and explained 7.9% of heterogeneity found (Supplementary Table 6). The funnel plot showed symmetry, suggesting no risk of publication bias (Supplementary Fig. 1). The full list of study-by-study rates and the description of complications are detailed in Supplementary Table 7.

**Table 1:** Pooled proportion (95% CI) of postoperative and intraoperative complications.

	*n*	Complication rate, %			Residual AKP	Residual PFI
		Overall*	Postoperative	Intraoperative		
Total derotational HTO	234	11.8 (6.6–18.0)^†^	9.1 (4.5–14.7)	4.2 (2.2–7.8)	18.2 (1.1–45.7)	0.0 (0.0–1.0)
Total DDFO	268	6.2 (1.0–14.1)	6.2 (1.0–14.1)	2.5 (1.0–5.8)	0.7 (0.0–4.9)	0.0 (0.0–0.3)
Total DLO	22	0.4 (0.0–14.3)	0.0 (0.0–3.2)	7.3 (1.5–3.0)	0.0 (0.0–3.2)	0.0 (0.0–3.2)
Total MD: HTO + DDFO	35	8.3 (0.7–21.0)	5.3 (0.0–16.6)	4.2 (0.8–18.3)	27.3 (0.0–98.9)	16.2 (0.0–73.0)
Total all	559	7.5 (3.9–11.8)^†^	5.9 (2.7–9.8)	3.8 (2.4–6.0)	7.6 (0.7–18.8)	0.1 (0.0–1.7)

*Combines intra- and post-operative complications; ^†^This rate includes one knee with complications not classifiable by the Clavien–Dindo scale (difficulty climbing stairs and pseudolocking). AKP, anterior knee pain; DDFO, derotational distal femoral osteotomy; DLO, double-level osteotomies; HTO, high tibial osteotomy; MD, mixed derotational; PFI, patellofemoral instability.

Knee stiffness (1.6%), local irritation (2.0%) and non-union (1.4%) were the most common postoperative complications. The larger portion of the reported postoperative complications was classified as grade IIIB (5.4%), primarily due to local irritation or non-union (Supplementary Table 8). Other complications were categorised as grade I (1.1%) and grade II (1.8%). No grade IIIA, IV or V complications were reported. Intraoperative complications were identified in 1.6% of the procedures, with the most common being injury to the peroneal nerve (0.7%). The proportion of overall postoperative complications (without residual AKP and PFI) was 5.9% (95% CI: 2.7–9.8%; *I*^2^ = 45) and was comparable in derotational HTO and DDFO (Table 1 and Supplementary Figure 2). Postoperative AKP was identified in 7.6% (95% CI: 0.7–18.8%; *I*^2^ = 94%) and residual PFI in 0.1% (95% CI: 0.0–1.7%; *I*^2^ = 48%). The proportion of AKP was more prevalent in derotational HTO than in DDFO (Table 1 and Supplementary Figs 3 and 4).

The pooled proportion of minor complications was 5.9% (95% CI: 2.0–10.9%; *I*^2^ = 52%), most commonly encompassing knee stiffness (*n* = 14) and local irritation (*n* = 11). Major complications were less common (6.4%, 95% CI: 3.4–10.0%; *I*^2^ = 0%; Supplementary Fig. 5) and included mostly non-union (*n* = 8) and peroneal nerve injury (*n* = 7).

Intraoperative complications were reported in a pooled proportion of 3.8% (95% CI: 2.4–6.0%; *I*^2^ = 0%; Table 1 and Supplementary Fig. 6). No intraoperative complications were reported for DDFO, and the most common intraoperative complication during derotational HTO was peroneal nerve injury (Supplementary Tables 6 and 8).

### Reinterventions, revisions and conversions to TKA

**Figure 4:**
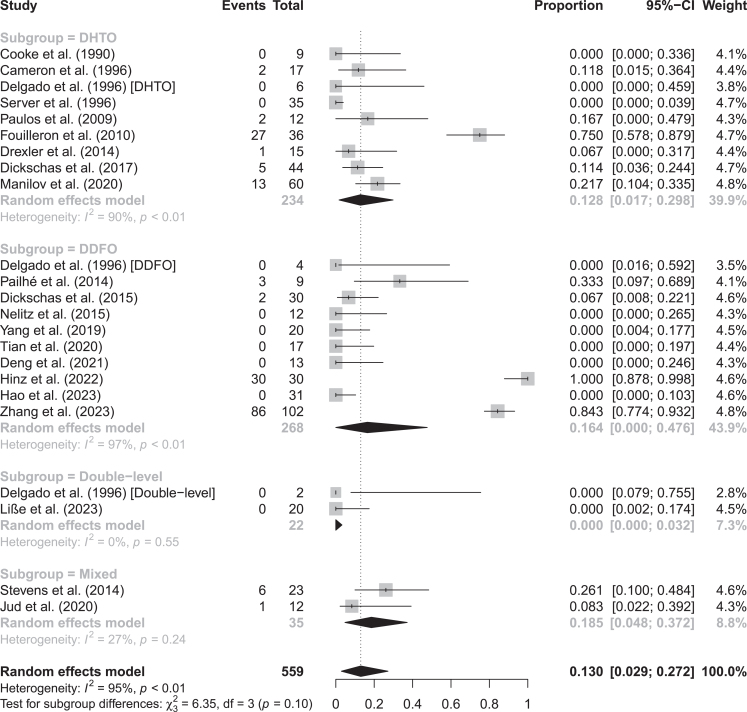
Forest plot on proportions of reinterventions subgrouped by the level of osteotomy (28, 29, 30, 31, 32, 33, 34, 35, 36, 37, 38, 39, 40, 41, 42, 43, 44, 45, 46, 47, 48).

The overall proportion of knees requiring reinterventions was 13.0% (95% CI: 2.9–27.2%; *I*^2^ = 95%), primarily for hardware removal (*n* = 158; 28.3%), correction of non-union (*n* = 8; 1.4%) and knee stiffness (*n* = 4; 0.7%). There were no differences in the proportion of reinterventions based on the level of knee derotational osteotomy (Table 2 and Fig. 4). Meta-regression showed that derotational HTO had an increasing effect on the proportion of reinterventions but did not explain any of the heterogeneity found (Supplementary Table 7). The funnel plot showed asymmetry suggesting risk of publication bias (Supplementary Fig. 5).

**Table 2:** Reinterventions, revisions and conversion to TKA.

Study	Reintervention rate, *n* (%)	Revision rate, *n* (%)	Reasons for revision/reintervention	Conversion to TKA rate, *n* (%)
Derotational HTO				
Cooke *et al.* (36)	0/9 (0)	0/0 (0)	NA	0 (0%)
Cameron *et al.* (37)	2/17 (11.8)	2/2 (100)	Hardware removal due to pain: 2	0 (0%)
Delgado *et al.* (38)	0/6 (0)	0/0 (0)	NA	0 (0%)
Server *et al.* (39)	0/35 (0)	0/0 (0)	NA	0 (0%)
Paulos *et al.* (40)	2/12 (16.7)	1/2 (49)	Knee stiffness correction: 1 (4 months postoperative); hardware removal: 1 (18 months postoperative)	0 (0%)
Fouilleron *et al.* (29)	27/36 (75)	25/27 (92.6)	Treatment of DVT which was found to be secondary to comprehension by the fibular fibrous arch: 1; knee stiffness correction: 1; hardware removal: 25 (27 ± 7.4 months postoperative)	0 (0%)
Drexler *et al.* (35)	1/15 (6.7)	1/1 (100)	Non-union correction: 1	1 (6.7%)*
Dickschas *et al.* (32)	5/44 (11.4)^†^	2/5 (39)	Non-union correction: 2; compartment syndrome treatment: 3 (the same patient required 3 surgeries for full recovery)	0 (0%)
Manilov *et al.* (45)	13/60 (21.7)	11/13 (84.6)	Knee stiffness correction: 2; hardware removal due to local irritation: 11	0 (0%)
DDFO				
Delgado *et al.* (38)	0/4 (0)	0/0 (0)	NA	0 (0%)
Pailhé *et al.* (33)	3/9 (33.3)	3/3 (100)	Non-union correction: 1; hardware removal due to discomfort: 2	0 (0%)
Dickschas *et al.* (41)	2/30 (6.7)	2/2 (100)	Non-union correction: 2 (4 and 7 months postoperative)	0 (0%)
Nelitz *et al.* (34)	0/12 (0)	0/0 (0)	NA	0 (0%)
Yang *et al.* (43)	0/20 (0)	0/0 (0)	NA	0 (0%)
Tian *et al.* (46)	0/17 (0)	0/0 (0)	NA	0 (0%)
Deng *et al.* (47)	0/13 (0)	0/0 (0)	NA	0 (0%)
Hinz *et al.* (28)	30/30 (100)	30/30 (100)	Hardware removal: 27; reosteosyntheses due to implant failure: 3	0 (0%)
Hao *et al.* (48)	0/31 (0)	0/0 (0)	NA	0 (0%)
Zhang *et al.* (30)	86/102 (84.3)	86/86 (100)	Hardware removal: 86	0 (0%)
Double-level derotational osteotomies				
Delgado *et al.* (38)	0/2 (0)	0/0 (0)	NA	0 (0%)
Liße *et al.* (31)	0/20 (0)	0/0 (0)	NA	0 (0%)
Mixed derotational HTO and DDFO				
Stevens *et al.* (42)	6/23 (26.1)	2/6 (33.3)	Non-union correction: 1; hardware removal and peroneal neurolysis in a patient with peroneal nerve irritation: 1; arthroscopic microfracture of patella: 1; isolated distal femoral hemiepiphysiodesis: 1; distal femoral hemiepiphysiodesis + knee arthroscopy + MPFLR: 1; TTT and LRR: 1	0 (0%)
Jud *et al.* (44)	1/12 (8.3)	1/1 (100)	Non-union correction: 1	0 (0%)
Pooled proportions (%, 95% CI)				
Derotational HTO	12.8 (1.7–29.8)	11.2 (1.3–26.5)		
DDFO	16.4 (0.0–47.6)	16.4 (0.0–47.6)		
Double-level	0.0 (0.0–3.2)	0.0 (0.0–3.2)		
Mixed	18.5 (4.8–37.2)	18.5 (4.8–37.2)		
Overall	13.0 (2.9–27.2)	12.3 (2.6–26.1)		

*Due to collapse at the tibial osteotomy site (6 months postoperatively); ^†^5 out of 49 knees were lost to follow-up. CI, confidence interval; DDFO, derotational distal femoral osteotomy; DVT, deep vein thrombosis; HTO, high tibial osteotomy; LRR, lateral retinacular release; MPFLR, medial patellofemoral ligament reconstruction; NA, not applicable; TTT, tibial tubercle transfer; TKA, total knee arthroplasty.

There was a pooled proportion of knees requiring revision procedures of 12.3% (95% CI: 2.6–26.1%; *I*^2^ = 95%) and without significant differences between subgroups based on the level of osteotomy (Table 2 and Supplementary Fig. 8). Revisions were mostly due to hardware removal.

When excluding the cases with potentially planned hardware removal, the pooled reintervention proportion was 3.2% (95% CI: 0.6–7.0%; *I*^2^ = 27%) and pooled revision proportion was 2.7% (95% CI: 0.3–6.5%; *I*^2^ = 64%) as reported in Supplementary Table 5.

Conversion to TKA was necessary in only one case (of derotational HTO) due to collapse at the osteotomy site.

## Discussion

The most important finding of this systematic review is that DDFOs and derotational HTOs may be used to treat AKP and/or PFI with a relatively low proportion of persistent pain (7.6%), almost no cases of residual instability (0.1%) and with moderately high incidence overall intra- and post-operative complication rates (7.5%). The rate of reinterventions was high, with 13.0% of patients needing a reintervention, mostly due to hardware removal. However, removing the cases of potentially planned hardware removal, the proportion of knees requiring reintervention (3.2%) and revision surgery (2.7%) was low. Our results must be taken into consideration with some caution due to the high risk of selection, performance and detection bias in nearly all studies.

Compared to a similar existing systematic review conducted by Sanchis-Alfonso *et al.* (49), a key difference is the inclusion of proximal femoral and distal tibial derotational osteotomies in their study. In contrast, this systematic review focuses only on derotational osteotomies around the knee, allowing a more in-depth summary of complications in this specific anatomical region. The current systematic review also includes a more complete description of complications and the evaluation of revision procedures and conversion to TKA. By addressing these factors, our systematic review fills an important gap in the existing body of knowledge, providing orthopaedic surgeons with a more detailed understanding of the associated risks to knee derotational osteotomies and deeper insights into the management of more complex complications. Finally, the previous systematic review failed to judge the risk of bias assessment, which is a cornerstone step in any systematic review to evaluate the internal validity of the included studies that may be under- or over-estimating the study findings.

### What is the risk profile of complications after knee derotational osteotomy?

Regarding the safety of this procedure, our findings revealed that 5.9% of the knees had minor complications and a higher proportion of major complications (6.4%). When considering both intra- and post-operative complications, the overall complication rate was 7.5%. It is essential to consider that a subset of patients may experience multiple complications (in the same knee) and that knee derotational osteotomy is a major surgery, typically reserved for more complex and uncommon clinical scenarios.

Residual PFI was rare (0.1% of the knees), and persistent AKP was more frequent (7.6%), but with most patients finding relief of their AKP during the follow-up period. This finding supports the hypothesis that realigning the knee rotational axis can resolve, in most cases, the AKP caused by rotational malalignment (either excessive femoral anteversion, tibial rotation or both). Remarkably, among the 21 included studies, only two cases of subsequent patellar dislocation were recorded (28, 31). It is important to recognise that AKP and PFI do not solely arise from the lower limb rotational malalignment and that there are other underlying causes (mostly anatomical) that need to be concomitantly addressed to present surgical failure and may have contributed to the lower risk of residual PFI and persistent AKP.

The most common postoperative complication observed was knee stiffness, which occurred in various degrees of severity, requiring intensive physiotherapy or arthroscopic adhesion debridement and manipulation under general anaesthesia. The hypothesis is that one of the variables influencing knee stiffness is the fact that the sectionised bones were fixed using a locking plate (29, 34, 44, 45, 46) or lateral compression staples (40). The use of lateral locking plates, characterised by their extended lever arm compared to intramedullary nails, poses challenges to immediate weight-bearing safety, potentially impacting bone healing and contributing to knee stiffness (50).

Within the spectrum of grade IIIB complications, the most common is non-union at the osteotomy site, all requiring revision that resulted in successful bone fusion. The union of the osteotomy is easier in the proximal region of the tibial tuberosity, where there is a higher percentage of trabecular bone (49). Nevertheless, it was not possible to have a substantial assessment of the comparative risk between DDFO and derotational HTO. Other contributing factors that may have affected the non-union include smoking and obesity (32, 35). Increasing age may also be a predisposing factor for non-union as this complication was more frequently reported in older ages (32, 35, 41). The choice of surgical technique may also impact the risk of non-union. For example, a lateral hinge can provide better stability; however, it is incompatible with derotational osteotomies as these procedures involve a full bone cut (41). Furthermore, fibular osteotomy is necessary in some specific cases to address fibular resistance to tibial rotation, carrying an increased risk of non-union (32).

### Which level of derotational osteotomy yields a higher proportion of complications?

When comparing the level of derotational knee osteotomy, the proportion of complications was higher in derotational HTO (11.8%) as compared to DDFO (6.2%), albeit without statistical significance. Although minor grade II complications (such as knee stiffness and knee joint clicking) were more frequent in DDFO, these complications are less clinically relevant. Importantly, no intraoperative complications were identified when the derotational osteotomy was performed at the distal femur level. Although the surgical indication for derotational HTO and DDFO will change according to the level of rotational malalignment (if excessive femoral anteversion or tibial rotation), the findings of this systematic review can inform the orthopaedic surgeon that a higher risk of complications should be expected when the derotational osteotomy is performed at the proximal tibia.

### Do concomitant or previous knee surgeries have any impact on the proportion of postoperative complications?

The addition of knee derotational osteotomy to soft tissue procedures often contributes to improving the overall outcomes of patellofemoral surgeries. There can be an increased risk of failure and, subsequently, residual AKP and PFI when torsional malalignments are left untreated (for instance, increasing the stress on the reconstructed MPFL (51, 52)). In fact, adding the derotational osteotomy to MPFL reconstruction or TTT has been shown to improve the postoperative outcomes (48, 53).

Given the multifactorial nature of AKP and PFI, it is essential to recognise that no singular procedure can be universally applicable in all cases, and treatment of complex cases of patellofemoral disorders needs to be *à la carte*. In our systematic review, 19 studies reported concomitant procedures alongside the derotational osteotomy, primarily MPFL reconstruction (≥33%) and lateral retinaculum release (29%). Considering these findings, residual PFI when performing concomitant surgeries is rare.

On the other hand, previous knee surgeries may have an impact on the risk of postoperative complications. In fact, a high number of previous surgeries may indicate that there is a complex underlying and unresolved patellofemoral disorder. This systematic review found that a significant proportion of knees had previously undergone lateral retinaculum release (≥5.9%) and TTT (≥5.1%), suggesting that these surgeries were unable to successfully treat the underlying problem. Dickschas *et al*. (41) and Cameron *et al*. (37) have shown that postoperative outcomes tend to be less favourable when prior surgery was performed, and Stevens and coworkers (42) ascribed the persistent AKP to the higher rate of previous surgeries. However, the studies that excluded patients with previous knee surgeries did not show a lower rate of complications (32, 43, 48).

### Is the risk of intraoperative complications a concern?

Only 3.8% of cases had intraoperative complications, and no intraoperative complications were reported for DDFO. The peroneal nerve injury was the most common, but the rate is even lower compared to lateral HTO, which yields a peroneal nerve injury in 3.2% of cases (54). The peroneal nerve involvement may have occurred due to factors such as nerve overextension, entrapment resulting from internal tibial rotation or fibular neck osteotomy. Interestingly, Manilov *et al*. (45) found that in their study, the nerve complications exclusively occurred when they performed the fibular osteotomy at the neck and the junction of the proximal two-thirds with the distal one-third of the fibula. This emphasises the importance of meticulous attention during the fibular neck osteotomy due to the nerve’s anatomical location. Fouilleron *et al.* (29) suggested to begin the surgical procedure at the fibular head and continuing towards the tubercle to protect the common peroneal nerve and to more safely perform the osteotomy of the fibular neck (when needed). Other reasons for peroneal nerve irritation may be related to loosening of the proximal tibial interlocking screw (42), which can be later treated with hardware removal and peroneal neurolysis. The remaining studies did not mention the possible reasons for peroneal nerve injury. Due to the high relevance of an iatrogenic peroneal nerve injury that can lead to paralysis and subsequent foot drop, orthopaedic surgeons must be cautious in approaching the joint to identify the peroneal nerve and prevent its injury. Liße *et al.* (31) suggested that the maximum internal correction should not exceed 15° to avoid damage to the peroneal nerve.

### Are we safe about the risk of revision and conversion to TKA?

Of the 559 knee derotational osteotomies, 12.3% required revision surgery with a higher incidence in the DDFO group (16.4%) than in the derotational HTO (11.2%). This is an interesting finding as although derotational HTO had a higher rate of complications, the complications were less severe and not requiring frequent reinterventions. The high rate of reinterventions in DDFO is coming from two studies (28, 30) in which 86% of cases required hardware removal, but the same was seen in derotational HTO with one study (29) reporting hardware removal in 69% of cases. When removing the cases of potentially planned hardware removal, the revision rates plumbed to 2.7%. The most common reason for unplanned removal of surgical hardware was local pain or irritation. However, it is important to note that hardware removal, while relatively frequent in knee osteotomy procedures (21% for TTT (55) and 10% for HTO (54)), may not hold significant clinical relevance as it is a common minor revision in these knee osteotomy procedures and should thus not be rigidly interpreted.

Only one case of conversion to TKA was reported, due to the unexpected collapse of the osteotomy site in a 57-year-old patient with a long history of PFI and a prior unsuccessful TTT (35). Although it is an encouraging low rate, it must be considered that the patients in the present systematic review were often young and that follow-up was not very long. In fact, TKA with derotational HTO is frequently indicated for an older population with severe torsion and PFI associated with osteoarthritis (56).

### Limitations

It is important to highlight some potential limitations inherent to the conducted systematic review. We only included studies written in English language, which resulted in a language bias. There was no available study comparing DDFO against derotational HTO, thus precluding a traditional comparative meta-analysis. Future studies should be conducted comparing these two different derotational osteotomies to ascertain which has the higher risk of complications by computing the relative risk between the two surgical approaches. Different surgeons, each with their surgical expertise and preferences, used different approaches, which resulted in variability in surgical techniques used that could introduce heterogeneity in the complication rates. There were two studies that reported a mixed sample using DDFOs and/or derotational HTOs without distinguishing between the two surgeries, not allowing to pinpoint the complication rates for each specific surgery (42, 44). Future studies should clarify when a DDFO and a derotational HTO are indicated. The high frequency of concomitant surgical interventions presents a substantial limitation, making it difficult to ascertain which precise surgical procedure primarily contributes to the observed complications (whether the derotational osteotomy, the concomitant surgery or the combination of both).

It is also important to acknowledge that the mean follow-up duration of approximately 46 months may not have provided a comprehensive assessment of the long-term risk of complications, especially because some might manifest only later in the patient’s recovery trajectory. This is particularly relevant for the outcome of conversion to TKA. One last consideration worth noting is that two studies (32, 42) had a complication rate over 100% due to multiple complications in the same knee, which affected the overall complication rate.

## Conclusions

Our findings underscore the role of DDFO and derotational HTO in resolving AKP and PFI in patients with torsional abnormalities. However, the rate of complications is moderately high and should not be undervalued. Notably, knee stiffness was the most common complication, along with hardware-related issues, non-union and peroneal nerve involvement. Although these complications were relatively infrequent, they underscore the need for vigilant intra- and postoperative monitoring and prompt intervention when necessary. Iatrogenic injury to the peroneal nerve can occur during derotational HTO surgery, and thus, orthopaedic surgeons must be cautious when approaching the joint to prevent the peroneal nerve injury.

## Supplementary materials



## ICMJE Conflict of Interest Statement

The authors declare that there is no conflict of interest that could be perceived as prejudicing the impartiality of the work.

## Funding Statement

This work did not receive any specific grant from any funding agency in the public, commercial or not-for-profit sector.

## Author contribution statement

All authors were involved in both the idealisation of the systematic review and preparation of the manuscript. IF and RA were involved in the database searches and data extraction. IF and RA performed data collection and organisation in coordination with RR and BF. IF performed all data analysis and interpretation of results in coordination with RA, EG and CV. IF and RA judged the risk of bias of the studies included in the systematic review. JEM, DD and MS guided and provided advice during all steps of the development of the systematic review. All authors contributed to drafting and approving the final manuscript prior to submission to the peer-reviewed journal.
