# Supplementary material for: Complications after knee derotational osteotomies in patients with anterior knee pain and/or patellofemoral instability: a systematic review with meta-analysis
Source: EFORT Open Rev. 2025 Jan 3;10(1):14–27. doi: 10.1530/EOR-2024-0036 (PMC11728914; doi:10.1530/EOR-2024-0036)
Supplement: Supplementary file 1 [file supplementary_materials.pdf]

List of Supplements

Supplementary Text 1 – Data collection and extraction details.

Supplementary Text 2 - Appraisal risk of bias criteria defined according to RoBANS tool.

Supplementary Table 1 - Search strategy for each database.

Supplementary Table 2 – Risk of bias criteria defined according to RoBANS tool.

Supplementary Table 3 - Characteristics of the studies and population.

Supplementary Table 4 - Surgical details.

Supplementary Table 5 – Sensitivity analyses excluding studies potential confounding factors.

Supplementary Table 6 – Reasons and rates of complications per individual study.

Supplementary Table 7 – Associations of study characteristics (age, sex and cell/tissue type) with intervention effects on TL (meta-regression).

Supplementary Table 8 – Overall incidence of complications (intra and postoperative).

Supplementary Figures

Reference list of all supplements

Supplementary Text 1 – Data collection and extraction details.

The following data was defined a prior (before systematic review initiation) and is below organised according to their domain and specific data collected:

Study characteristics: study reference (author, year), country where the study was carried out, sample size, and follow-up;

Patients metrics: age, sex (male or female), body mass index (BMI), number of knees, knee laterality, diagnosis, and previous knee surgeries;

Intervention: type of osteotomy performed (distal femoral or proximal tibial) and concomitant surgeries;

Outcomes: rates and description of intra and postoperative complications (including residual AKP and PFI), reinterventions, revisions and conversions to TKA (with reasons included).

Supplementary Text 2 – Data management details.

Modified Clavien-Dindo scale for complications in orthopaedic surgery

Grade I complications are those that spontaneously resolve without the need for pharmacological treatment (except antipyretics, anti-diarrheal, antiemetics). Grade II complications require medication for more than 72 hours or active observation without interventions. Grade IIIA complications are resolved using local anaesthesia during the procedure, while Grade IIIB complications require surgery, endoscopy, or radiological interventions with general anaesthesia. Grade IV complications necessitate management in the intensive care unit, and Grade V means a fatal outcome. Peroneal nerve complications and fractures were categorised as intraoperative complications and were not subjected to classification according to this scale.

Definition of minor and major complications

Minor complications, for purposes of this systematic review, were defined as difficulty climbing stairs, clicking of the knee joint, knee stiffness, pseudolocking, local irritation, discomfort/pain at the osteotomy site, phlebitis, and delayed wound healing. Major complications encompassed non-union of the osteotomy site, peroneal nerve injury, fracture, compartment syndrome, and DVT.

Other data management procedures

In instances where studies did not provide explicit information regarding reinterventions, revisions and TKA conversions, it was assumed incidence rate of 0%; given the high relevance of conversion to TKA, it is expected that, when occurring, they were reported.

Overall postoperative and overall complication rates (intra and postoperative) were determined by combining the rate of all reported complications, without considering the residual AKP and PFI as complications. It was decided to assess the rate of complications based on the osteotomy level: (i) isolated “derotational HTO”; (ii) isolated “DDFO”; (iii) "mixed derotational HTO and DDFO" refers to groups that report complications of derotational HTO and DDFO without distinguishing between the specific levels of osteotomy; and (iv) "concomitant derotational HTO and DDFO" refers to groups that include double-level osteotomy procedures.

Supplementary Table 1 – Search strategy for each database.

Databases	Results	Search strategy	Date
PubMed	2624	(((((tibia OR tibial OR femur OR femoral OR knee) AND (osteotom*)) AND (rotational OR rotatory OR torsion OR torsional OR derotational OR desrotational OR misalignment))))	30/09/2023
EMBASE	1958	('tibia'/exp OR tibia OR tibial OR 'femur'/exp OR femur OR femoral OR 'knee'/exp OR knee) AND osteotom* AND (rotational OR rotatory OR 'torsion'/exp OR torsion OR torsional OR derotational OR desrotational OR misalignment)	30/09/2023
Web of Science	1709	(((((tibia OR tibial OR femur OR femoral OR knee) AND (osteotom*)) AND (rotational OR rotatory OR torsion OR torsional OR derotational OR desrotational OR misalignment))))	30/09/2023
**Total**	**6291**		

.

Supplementary Table 2 – Risk of bias criteria defined according to RoBANS tool.

Domain	Description
**Selection of participants**	**Selection bias** caused by inadequate selection of participants.<br><br>Participants should have experienced AKP or PFI resulting from lower limb rotational malalignments and undergone distal femoral and/or proximal tibial derotational osteotomy treatment.<br><br>The inclusion of individuals with AKP and PFI in the same sample is considered as high risk. The inclusion of individuals with AKP or PFI caused by factors other than rotational malalignments of the lower limb is considered as high risk. The inclusion of different age groups (paediatric, adolescent, and adult participants) in the same sample is considered as high risk.
**Confounding variables**	**Selection bias** caused by inadequate confirmation and consideration of confounding variables.<br><br>The participant or group samples should be homogenous in terms of confounding variables, such as:<br><br>- ***Patient-related factors****:* age, sex, concomitant knee injuries and the underlying causes of AKP or PFI.<br>- ***Intervention-related factors***: previous and concomitant surgical procedures, methods for derotational osteotomy and unclear distinction between distal femoral derotational osteotomies and proximal tibial derotational osteotomies when reporting complications.<br>- ***Outcome****:* time of follow-up.<br><br>When incorporating comparison groups (and considered for the systematic review), the groups must exhibit homogeneity with respect to confounding variables (*e.g.*, age, follow-up, previous surgeries, concomitant surgeries, among others).
**Exposure measurement**	**Performance bias** caused by inadequate measurement of exposure.<br><br>Data on complications, revisions, and conversions to arthroplasty must be collected prospectively. For complications, time to follow-up should be at least 12 months. For revisions and conversions to arthroplasty, the time to follow-up should be at least 24 months.<br><br>Complications must be described regarding their type and treatment performed. Studies that included AKP or PFI must report residual pain or instability, respectively. Studies that only report the number and/or rate of complications without describing them are judged with high risk.<br><br>Studies that refer revisions and conversions to arthroplasty must include a description of the reasons contributing to the failure of the initial surgical procedure. Studies that only report the number and/or rate of revisions or conversions to arthroplasty without providing reasons of failure are judged with high risk.
**Blinding outcome assessment**	**Detection bias** caused by inadequate blinding of outcome assessment.<br><br>Assessor and/or data analysist not blinded intervention when evaluating complications. However, high risk of detection bias was not considered for revision and conversion to arthroplasty because being aware of intervention has a low likelihood of biasing these outcomes.
**Incomplete outcome data**	**Attrition bias** caused by inadequate handing of incomplete data outcome.<br><br>Missing data or loss to follow-up in >5% of any outcome variable is judged with high risk.

**Selective outcome reporting**	**Reporting bias** caused by selective outcome reporting.<br><br>Based on reporting of the collected/assessed outcomes and statistical analysis, studies where the methods provided no information on how the analyses would be performed are judged with high risk. Studies with significant attrition bias and the reasons for missing data were unreported are judged with high risk.

Legend: AKP - Anterior Knee Pain; PFI - Patellofemoral Instability.

Supplementary Table 3 – Characteristics of the studies and population.

First author, year	Region	Number of patients (knees)	Age, mean ± SD (years)	Sex, n	BMI, mean ± SD (Kg/m^2)	Knee laterality, n	Condition/diagnosis
**Cooke et al. (1990) (1)**	Canada	12 (9) *	18 (one patient was 46 years old)	3M:9F	NR	NR	AKP
**Cameron et al. (1996) (2)**	Canada	16 (17)	27.6 (14-42)	0M:16F	NR	Left: 8<br>Right: 9	Recurrent patellar dislocation: all cases<br>AKP: 5 cases
**Delgado et al. (1996) (3)**	USA	5 (12)	15.2 ± 2.59	4M:1F	NR	Left: 6<br>Right: 6	AKP
**Server et al. (1996) (4)**	Spain	25 (35)	20 (15-45)	3M:22F	NR	NR	AKP: all cases<br>PFPS: 26 cases<br>PFI: 9 cases<br>Recurrent patellar dislocation: 2 cases
**Paulos et al. (2009) (5)**	USA	12 (12)	20 (15–30)	3M:9F	NR	NR	PFI: all patients (average number of episodes: 8.6)
**Fouilleron et al. (2010) (6)**	France	29 (36)	26.5 ± 7.4	5M:24F	NR	Left: 18<br>Right: 18	PFI: 5 cases<br>PFPS: 31 cases
**Drexler et al. (2014) (7)**	Canada	12 (15)	34.6 † (19-57)	1M:11F	N.R.	NR	PFI
**Pailhé et al. (2014) (8)**	France	6 (9)	13.6 ± 1.9	2M:4F	18.4 ± 2.5	NR	AKP
**Stevens et al. (2014) (9)**	USA	16 (23)	17 (9-30)	3M:13F	NR	Left: 10<br>Right: 13	AKP and/or PFI
**Dickschas et al. (2015) (10)**	Germany	25 (30)	30.5 (15-47)	6M:19F	NR	NR	Patellar dislocation: 15 cases<br>AKP: 17 cases
**Nelitz et al. (2015) (11)**	Germany	12 (12)	18.2 (15–26)	0M:12F	NR	NR	Recurrent patellar dislocation (≥2 times)
**Dickschas et al. (2017) (12)**	Germany	42 (49)	27 ± 9.79	13M:29F	NR	NR	AKP or PFI
**Yang et al. (2019) (13)**	China	20 (20)	21 ± 4.2	2M:18F	25.2 ± 4.2	NR	Recurrent patellar dislocation (≥3 times)
**Jud et al. (2020) (14)**	Switzerland	10 (12)	24 (15-40)	0M:10F	NR	Left: 6<br>Right: 6	PFI

**Manilov et al. (2020) (15)**	Argentina	60 (60)	30.5 (18-61)	10M:50F	<25: 42p<br>25-30: 13p<br>>30: 5p	Left: 31<br>Right: 29	AKP
**Tian et al. (2020) (16)**	China	16 (17)	20.8 ± 6.58	5M:11F	24.2 ± 2.7	Left: 7<br>Right: 10	Patellar dislocation (≥2 times): all<br>AKP: 16 cases
**Deng et al. (2021) (17)**	China	13 (13)	18.7 (15-29)	4M:9F	25.7 ± 3.6	Left: 5<br>Right: 8	Recurrent patellar dislocation (≥2 times)
**Hinz et al. (2022) (18)**	USA	27 (30)	23.5 † (19.8-29.0)	4M:23F	24.3 ± 4.7	NR	Recurrent patellar instability
**Hao et al. (2023) (19)**	China	31 (31)	23.7 ± 8.9	4M:27F	25.6 ± 3.8	Left: 17<br>Right: 14	Recurrent patellar dislocation (≥2 times)
**Liße et al. (2023) (20)**	Germany	18 (20)	23.1 ± 7.39	2M:16 F	21.2 ± 3.3	Left: 7<br>Right: 13	AKP: all cases<br>Patellar dislocation: 9 cases
**Zhang et al. (2023) (21)**	China	92 (102)	21.6 ± 6.9 (15-46)	8M: 94 F§	21.4 ± 2.6	Left: 46<br>Right: 56	Recurrent patellar dislocation

Legend: AKP - Anterior Knee Pain; BMI - Body Mass Index; F – female; M – male; n - sample size; NR - Not Reported; PFI - Patellofemoral Instability; PFPS - Patellofemoral Pain Syndrome; SD - Standard Deviation; USA - United States of America.

* Only 7 patients (9 knees) of the 12 patients underwent osteotomy. Age and sex are described for the 12 patients.

† Median value

‡ The grading method was as follows: grade 1- 1 quadrant of motion; grade 2- 2 quadrants of motion; and grade 3- complete patellofemoral dislocation.

§ Sex distribution was related (in original publication) to the number of knees and not number of patients.

Supplementary Table 4 – Surgical details.

First author, year	Type of procedure	Previous unsuccessful knee surgeries	Follow-up, mean ± SD (months)
**Cooke et al. (1990) (1)**	Derotational HTO combined with concomitant LRR	LRR, medial plication or the Elmslie-Trilat procedure: 6 knees	>24
**Cameron et al. (1996) (2)**	Derotational HTO<br><br>Concomitant procedures:<br>Maquet type osteotomy: 5 knees	LRR: 5 knees<br>Hauser procedure: 5 knees<br>Maquet procedure: 3 knees<br>Medialization of the patellar tendon: 3 knees<br>Semitendinosus tenodesis: 1 knee	25 (12-38)
**Delgado et al. (1996) (3)**	Isolated DDFO: 4 knees<br>Isolated derotational HTO: 6 knees<br>Double-level osteotomy: 2 knees	NR	32.8 ± 12.5
**Server et al. (1996) (4)**	Derotational HTO combined with concomitant LRR	Soft tissue procedures: 2 knees	51.6 (12-96)
**Paulos et al. (2009) (5)**	Derotational HTO combined with TTT	Range of prior operations = 0-7 knees	Range: 36-48
**Fouilleron et al. (2010) (6)**	Derotational HTO<br><br>Concomitant lateral wedge resection when necessary.	TTT: 3 knees<br>LRR: 3 knees<br>Maquet technique: 2 knees	55 (12-174)
**Drexler et al. (2014) (7)**	Derotational HTO combined with TTT	TTT + LRR + medial plication: 10 knees<br>Isolated soft tissue corrections: 5 knees	84 (median)<br>(15-156)
**Pailhé et al. (2014) (8)**	DDFO	NR	23 ± 8.5
**Stevens et al. (2014) (9)**	Isolated DDFO: 1 knee<br>Isolated derotational HTO: 14 knees<br>Double-level osteotomy: 8 knees<br><br>Concomitant LRR: 8 knees	-TTT: 12 knees<br>-Arthroscopic debridement: 11 knees<br>-LRR: 5 knees<br>-Medial reefing: 2 knees<br>-MPFLR: 1 knee<br>-Microfracture: 1 knee	59.2 (11-145)
**Dickschas et al. (2015) (10)**	DDFO<br><br>Concomitant procedures:<br>-Valgisation osteotomy: 8 knees<br>-Varisation osteotomy: 6 knees<br>-Medial retinaculum plasty: 5 knees<br>-Plica resection: 3 knees<br>-Microfracture: 3 knees	-Medial retinaculum plasty: 12 knees<br>-TTT: 3 knees<br>-Trochleoplasty: 2 knees<br>-Chondroplasty: 2 knees<br>-LRR: 2 knees	41 (6-113)

	-LRR: 3 knees<br>-TTT: 3 knees<br>-Notchplasty: 2 knees<br>-Extension: 2 knees<br>-Removal of an osteochondral joint body: 1 knee<br>-Partial resection of the meniscus: 1 knee		
**Nelitz et al. (2015) (11)**	DDFO combined with MPFLR	Medial reefing: 2 knees	16.4 (12-28)
**Dickschas et al. (2017) (12)**	Derotational HTO combined with LRR<br><br><u>Concomitant procedures:</u><br>- MPFLR in an undisclosed number of cases<br>- Valgisation osteotomy: 21 knees<br>- Plica resection: 16 knees<br>- Smoothing of cartilage: 7 knees<br>- Fibular osteotomy: 7 knees<br>- Tightening of the medial retinaculum: 4 knees<br>- Microfracture: 4 knees<br>- Medial retinaculum plasty: 2 knees<br>- Refixation of an osteochondral fragment: 2 knees<br>- Resection of medial patellar fragments: 1 knee<br>- Resection of the lateral patellar facet: 1 knee<br>- Removal of an osteochondral joint body:1 knee<br>- Refixation of the meniscus: 1 knee	None of the knee had previous knee surgery	42 (6-131)
**Yang et al. (2019) (13)**	DDFO combined with medial retinaculum plasty	None of the knee had previous knee surgery	18 (12-23)
**Jud et al. (2020) (14)**	Isolated DDFO: 5 knees<br>Double-level osteotomy: 7 knees<br><br><u>Concomitant procedures:</u><br>-MPFLR: 11 knees<br>-Trochleoplasty: 7 knees<br>-TTT: 4 knees<br>-Lengthening of the lateral retinaculum: 3 knees	NR	11 ± 6
**Manilov et al. (2020) (15)**	Derotational HTO<br><br>Concomitant LRR: 46 knees	LRR: 8 knees<br>Partial meniscectomy: 2 knees<br>Medial capsular plication: 1 knee<br>TTT: 1 knee	66 (24-157)

**Tian et al. (2020) (16)**	DDFO<br><br>Concomitant procedures:<br>-Medial retinaculum plasty: 5 knees<br>-MPFLR: 4 knees	NR	26.5 (12-49)
**Deng et al. (2021) (17)**	DDFO combined with MPFLR	NR	26.7 (24-33)
**Hinz et al. (2022) (18)**	DDFO<br><br>Double-level osteotomy: 1 knee<br><br>Concomitant procedure:<br>-MPFLR: 21 knees<br>-Varization osteotomy: 14 knees<br>-Lateral patellar retinacular lengthening: 3 knees<br>-Trochleoplasty: 3 knees<br>-Patellar lateral facetectomy: 2 knees<br>-TTT: 1 knee<br>-Vastus medialis oblique transfer: 1 knee<br>-Medial closing-wedge osteotomy:1 knee	Previous surgeries for PFI: 13 knees	38.0 (median)<br>(31.8–52.5)
**Hao et al. (2023) (19)**	DDFO combined with MPFLR	None of the knee had previous knee surgery	43.4 ± 9.4
**Liße et al. (2023) (20)**	Double-level osteotomy: all<br><br>Concomitant procedures:<br>-Valgisation osteotomy: 2 knees<br>-MPFLR: 2 knees<br>-Plica resection: 2 knees<br>-LRR: 1 knee	Medial retinaculum plasty: 2 knees<br>Femoral and tibial closing wedge valgisation osteotomy: 1 knee	59.1 (9-173)
**Zhang et al. (2023) (21)**	DDFO combined with MPFLR<br><br>Concomitant TTT: 23 knees	NR	49.2 ± 15.6

Legend: DDFO - Derotational Distal Femoral Osteotomy; HTO - High Tibial Osteotomy; LRR - Lateral Retinaculum Release; MPFLR - Medial Patellofemoral Ligament Reconstruction; NR - Not Reported; SD - Standard Deviation; TTT - Tibial Tubercle Transfer.

Supplementary Table 5 – Sensitivity analyses excluding studies potential confounding factors.

Outcome	Group / subgroup	Original findings		Sensitivity analyses		
		**Proportion (95% CI)**	**I, P value**	**Proportion (95% CI)**	**I^2**	**Reasons for exclusion and removed studies**
**Overall complications**	DDFO	6.2% (1.0% to 14.1%)	62.6%	7.7% (1.8% to 16.3%)	60.3%	Removed Hinz et al. (18) because it had one case of double level osteotomy mixed with DDFO.
	Total	7.5% (3.9% to 11.8%)	48.3%	8.3% (4.7% to 12.6%)	41.2%	
**Overall complications**	DDFO	6.2% (1.0% to 14.1%)	62.6%	1.5% (0.0% to 4.3%)	0.0%	Removed Pailhé et al. (8), Nelitz et al. (11) and Yang et al. (13) due to short follow-up (< 24 months)
	Mixed	8.3% (0.7% to 21.0%)	0.0%	8.7% (0.2% to 24.5%)	---	Removed Jud et al. (14) due to short follow-up (< 24 months)
	Total	7.5% (3.9% to 11.8%)	48.3%	5.7% (2.5% to 9.8%)	42.1%	Removed Pailhé et al. (8), Nelitz et al. (11), Yang et al. (13) and Jud et al. (14) due to short follow-up (< 24 months)
**Overall reinterventions**	DHTO	12.8% (1.7% to 29.8%)	89.8%	6.1% (1.7% to 12.2%)	62.4%	Removed cases of potentially planned hardware removal from studies Hinz et al. (18), Zhang et al. (21) and Fouilleron et al. (6).
	DDFO	16.4% (0.0% to 47.6%)	97.2%	1.0% (0.0% to 5.4%)	44.0%	
	Total	13.0% (2.9% to 27.2%)	95.1%	3.2% (0.6% to 7.0%)	27.1%	
**Overall reinterventions**	DDFO	16.4% (0.0% to 47.6%)	97.2%	22.0% (0.0% to 66.3%)	97.8%	Removed Pailhé et al. (8), Nelitz et al. (11) and Yang et al. (13) due to short follow-up (< 24 months)
	Mixed	18.5% (4.8% to 37.2%)	27.1%	26.1% (9.8% to 46.2%)	---	Removed Jud et al. (14) due to short follow-up (< 24 months)
	Total	13.0% (2.9% to 27.2%)	95.1%	14.6% (2.6% to 31.9%)	95.7%	Removed Pailhé et al. (8), Nelitz et al. (11), Yang et al. (13) and Jud et al. (14) due to short follow-up (< 24 months)
**Overall revision**	DHTO	11.2% (1.3% to 26.5%)	88.2%	4.6% (0.5% to 11.2%)	61.5%	Removed cases of potentially planned hardware removal from studies Hinz et al. (18), Zhang et al. (21) and Fouilleron et al. (6).
	DDFO	16.4% (0.0% to 47.6%)	97.2%	1.0% (0.0% to 5.4%)	56.2%	
	Total	12.3% (2.6% to 26.1%)	95.0%	2.7% (0.3% to 6.5%)	64.3%	

Supplement Table 6 – Reasons and rates of complications per individual study.

First author, year	Overall intra- and postoperative complication rate, n (%) ‡‡	Postoperative complication description	Postoperative complications						Follow-up duration (months)	Residual AKP, n (%)	Residual PFI, n (%)	Intraoperative complications, n (%)
			Grade I	Grade II	Grade III A	Grade III B	Grade IV	Grade V				
Derotational HTO												
**Cooke et al. (1990) (1)**	1/9 (11.1%)	Delayed wound healing: 1	0	1	NR	NR	NR	NR	>24	NR	NA	NR
**Cameron et al. (1996) (2)**	2/17 (11.8%)	Staple pain at the osteotomy site: 2 (requiring revision)	0	0	0	2	0	0	25 (12-38)	1 (5.9%)	0 (0%)	0 (0%)
**Delgado et al. (1996) (3)**	0/6 (0.0%)	NA	0	0	0	0	0	0	32.8 ± 12.5	NR	NA	0 (0%)
**Server et al. (1996) (4)**	4/35 (11.4%)	Phlebitis: 1 (improved with standard treatment)<br><br>Difficulty climbing stair and pseudolocking:1 §§	1	NR	NR	NR	NR	NR	51.6 (12-96)	9 (25.7%) *	2 (5.7%)	Fracture without displacement: 1 (healed in 2 months of nonweightbearing)<br><br>Irritation of the sensory fibers of the peroneal nerve: 1 †
**Paulos et al. (2009) (5)**	1/12 (8.3%)	Knee Stiffness: 1 (requiring surgical correction)	0	0	0	1	0	0	Range: 36-48	3 (25%) ‡	0 (0%)	0 (0%)
**Fouilleron et al. (2010) (6)**	3/36 (8.3%)	DVT: 1 (requiring surgery)<br><br>Knee Stiffness: 1 (requiring surgical correction)	NR	NR	NR	2	NR	NR	55 (12-174)	20 (55.6%) §	0 (0%)	Transitory Peroneal Nerve Palsy: 1 (recovered naturally after 6 months)
**Drexler et al. (2014) (7)**	2/15 (13.3%)	Non-union: 2 (requiring revision; one in a heavy smoker and another in a patient with unsuccessful TTT)	NR	NR	NR	2	NR	NR	84 (median) (15-156)	NA	NR	NR
**Dickschas et al. (2017)**	4/44 (9.1%)	Non-union: 2 (requiring revision) ¶	0	0	0	3	0	0	42 (6-131)	42 (95.4%) #	0 (0%)	Transitory peroneal nerve palsy: 1 **

**(12)**		Compartment syndrome: 1 (requiring cleavage)										
**Manilov et al. (2020) (15)**	16/60 (26.7%)	Knee stiffness: 2 (requiring revision)<br><br>Local irritation: 11 (requiring revision)	0	0	0	13	0	0	66 (24-157)	NR	NA	Proximal tibial fracture distal to the osteotomy (fixed with longer plate during the same surgery): 1<br><br>Transitory peroneal nerve palsy (recovered naturally after 30 days): 1<br><br>Permanent peroneal nerve palsy: 1 ††
DDFO												
**Delgado et al. (1996) (3)**	0/4 (0.0%)	NA	0	0	0	0	0	0	32.8 ± 12.5	NR	NA	0 (0%)
**Pailhé et al. (2014) (8)**	3/9 (33.3%)	Non-union: 1 (requiring revision)<br><br>Discomfort at the osteotomy site: 2 (requiring revision)	0	0	0	3	0	0	23 ± 8.5	NR	NA	0 (0%)
**Dickschas et al. (2015) (10)**	2/30 (6.7%)	Non-union: 2 (requiring revision)	NR	NR	NR	2	NR	NR	41 (6-113)	NR	0 (0%)	NR
**Nelitz et al. (2015) (11)**	2/12 (16.7%)	Knee Stiffness: 2 (requiring a prolongated rehabilitation)	0	2	0	0	0	0	16.4 (12-28)	NA	0 (0%)	NR
**Yang et al. (2019) (13)**	6/20 (30%)	Knee stiffness: 1 (improved with passive exercises)<br><br>Occasional knee joint clicking during flexion: 2<br><br>Lateral thigh pain: 3 (gradually relieved with daily activity)	5	1	0	0	0	0	18 (12-23)	0 (0%)	0 (0%)	0 (0%)
**Tian et al. (2020) (16)**	2/17 (11.8%)	Knee stiffness: 2 (improved after half a month of passive	NR	2	NR	NR	NR	NR	26.5 (12-49)	2 (11.8%)	0 (0%)	NR

		exercise enhancement)										
**Deng et al. (2021) (17)**	0/13 (0.0%)	NA	0	0	0	0	0	0	26.7 (24-33)	NA	NR	0 (0%)
**Hinz et al. (2022) (18)**	1/30 (3.3%)	Patellar dislocation: 1	0	0	0	0	0	0	38.0 (median) (31.8–52.5)	NR	1 (3.3%)	0 (0%)
**Hao et al. (2023) (19)**	1/31 (3.2%)	Knee stiffness: 1 (resolved after 2 months of intensified rehabilitation)	NR	1	NR	NR	NR	NR	43.4 ± 9.4	6 (19.4%) ‡‡	0 (0%)	NR
**Zhang et. al (2023) (21)**	3/102 (2.9%)	Knee stiffness: 3 (resolved with prolongated rehabilitation)	0	3	0	0	0	0	49.2 ± 15.6	NA	0 (0%)	0 (0%)
Double-level derotational HTO and DDFO												
**Delgado et al. (1996) (3)**	0/2 (0%)	NA	0	0	0	0	0	0	32.8 ± 12.5	NR	NA	0 (0%)
**Liße et al. (2023) (20)**	1/20 (5%)	NA	0	0	0	0	0	0	59.1 (9-173)	NR	1 (5%)	Transitory peroneal nerve palsy (recovered nearly completely): 1
Mixed derotational HTO and DDFO												
**Stevens et al. (2014) (9)**	2/23 (8.7%)	Femoral non-union: 1	0	0	0	1	0	0	59.2 (11-145)	16 (70%)	10 (43.5%)	Peroneal nerve irritation (caused by a loose proximal tibial interlocking screw): 1
**Jud et al. (2020) (14)**	1/12 (8.3%)	Knee Stiffness: 1 (requiring revision)	0	0	0	1	0	0	11 ± 6	NR	NR	0 (0%)

Legend: AKP - Anterior Knee Pain; DDFO - Derotational Distal Femoral Osteotomy; DVT - Deep Venous Thrombosis; HTO - High Tibial Osteotomy; n - sample size; NA - Not applicable; NR - Not Reported; PFI - Patellofemoral Instability; TTT - Tibial Tubercle Transfer.

* Occasional pain on strenuous activity: 6 cases. Moderate pain: 2 cases. Pain on flexion and extension and disability: 1 case.
† Pain and paraesthesiae for several hours in the first web space and over the dorsum of the foot, but there was no paralysis.
‡ Occasional aching with changes in weather.
§ Minimal pain: 14 cases. Mild pain: 3 cases. Significant pain: 3 cases. Severe pain: 0 cases.
& Five out of forty-nine knees were lost to follow-up.
¶ One of them was fibular non-union.
# The study mentions that 35 out of 42 patients had residual AKP. Since counting the number of knees with AKP was not possible, was made a deduction to address this limitation.

** Without compartment syndrome showing paresthesia interdigital I/II and slight weakness of extension of the hallux.
†† Permanent palsy of the extensor hallucis longus.
§§ Not accounted for grading.
‡‡ Not accounting for residual AKP or persistent PFI.

Supplement 7 – Associations of study characteristics (age, sex and cell/tissue type) with intervention effects on TL (meta-regression).

Outcome	Factor	Intercept (95% CI)	ß values (95% CI)	*P* value	Heterogeneity (R^2)
**Overall complications**	Age	0.2717 (-0.0195 to 0.5628)	0.0026 (-0.0097 to 0.0148)	0.1	0.00%
	Duration of follow-up*	0.3471 (0.1810 to 0.5132)	-0.0004 (-0.0040 to 0.0033)	0.001	0.00%
	Level of osteotomy (DDFO)	0.2923 (0.2026 to 0.3820)	0.0864 (-0.0427 to 0.2155)	0.001	7.92%
	Level of osteotomy (Double-level)		-0.0189 (-0.2931 to 0.2553)		
	Level of osteotomy (Mixed)		0.0408 (-0.1879 to 0.2695)		
**Reinterventions**	Age	0.2176 (-0.4762 to 0.9114)	0.0085 (-0.0217 to 0.0388)	1.0	0.00%
	Duration of follow-up*	0.2375 (-0.1561 to 0.6311)	0.0043 (-0.0048 to 0.0134)	1.0	0.00%
	Level of osteotomy (DDFO)	0.4503 (0.1975 to 0.7031)	-0.0587 (-0.4251 to 0.3078)	0.001	0.00%
	Level of osteotomy (Double-level)		-0.2625 (-0.9273 to 0.4024)		
	Level of osteotomy (Mixed)		-0.0028 (-0.6204 to 0.6148)		

* When only reported as ranges, it was used the lower bound of the interval.

Note: Meta-regressions on level of osteotomy are being compared against derotational HTO (intercept).

Supplementary Table 8. Overall incidence of complications (intra and postoperative).

Complications	Derotational HTO (n=234)	DDFO (n=268)	Concomitant derotational HTO and DDFO (n=22)	Mixed derotational HTO and DDFO (n=35)	Total (n=559)
**Postoperative complications**					
Difficulty climbing stairs and pseudolocking	1 (0.4%)	0 (0%)	0 (0%)	0 (0%)	1 (0.2%)
**Grade I**					
Pain in the lateral thigh	0 (0%)	3 (1.1%)	0 (0%)	0 (0%)	3 (0.5%)
Clicking of knee joint	0 (0%)	2 (0.8%)	0 (0%)	0 (0%)	2 (0.4%)
Phlebitis	1 (0.4%)	0 (0%)	0 (0%)	0 (0%)	1 (0.2%)
**Grade II**					
Knee Stiffness	0 (0%)	9 (3.4%)	0 (0%)	0 (0%)	9 (1.6%)
Delayed wound healing	1 (0.4%)	0 (0%)	0 (0%)	0 (0%)	1 (0.2%)
**Grade III_B**					
Local irritation	11 (4.7%)	0 (0%)	0 (0%)	0 (0%)	11 (2.0%)
Non-union	4 (1.7%) *	3 (1.1%)	0 (0%)	1 (2.9%) †	8 (1.4%)
Knee Stiffness	4 (1.7%)	0 (0%)	0 (0%)	1 (2.9%)	5 (0.9%)
Discomfort/pain at the osteotomy site	2 (0.9%)	2 (0.8%)	0 (0%)	0 (0%)	4 (0.7%)
Compartment syndrome	1 (0.4%)	0 (0%)	0 (0%)	0 (0%)	1 (0.2%)
DVT	1 (0.4%)	0 (0%)	0 (0%)	0 (0%)	1 (0.2%)
**Intraoperative complications**					
Transitory peroneal nerve paralysis	3 (1.3%)	0 (0%)	1 (4.6%)	0 (0%)	4 (0.7%)
Fracture	2 (0.9%)	0 (0%)	0 (0%)	0 (0%)	2 (0.4%)
Peroneal nerve irritation	1 (0.4%)	0 (0%)	0 (0%)	1 (2.9%)	2 (0.4%)
Permanent peroneal nerve paralysis	1 (0.4%)	0 (0%)	0 (0%)	0 (0%)	1 (0.2%)

Legend: DDFO - Derotational Distal Femoral Osteotomy; DVT - Deep Venous Thrombosis; HTO - High Tibial Osteotomy.

* One is fibular non-union.

† One is femoral non-union.

SUPPLEMENTARY FIGURES

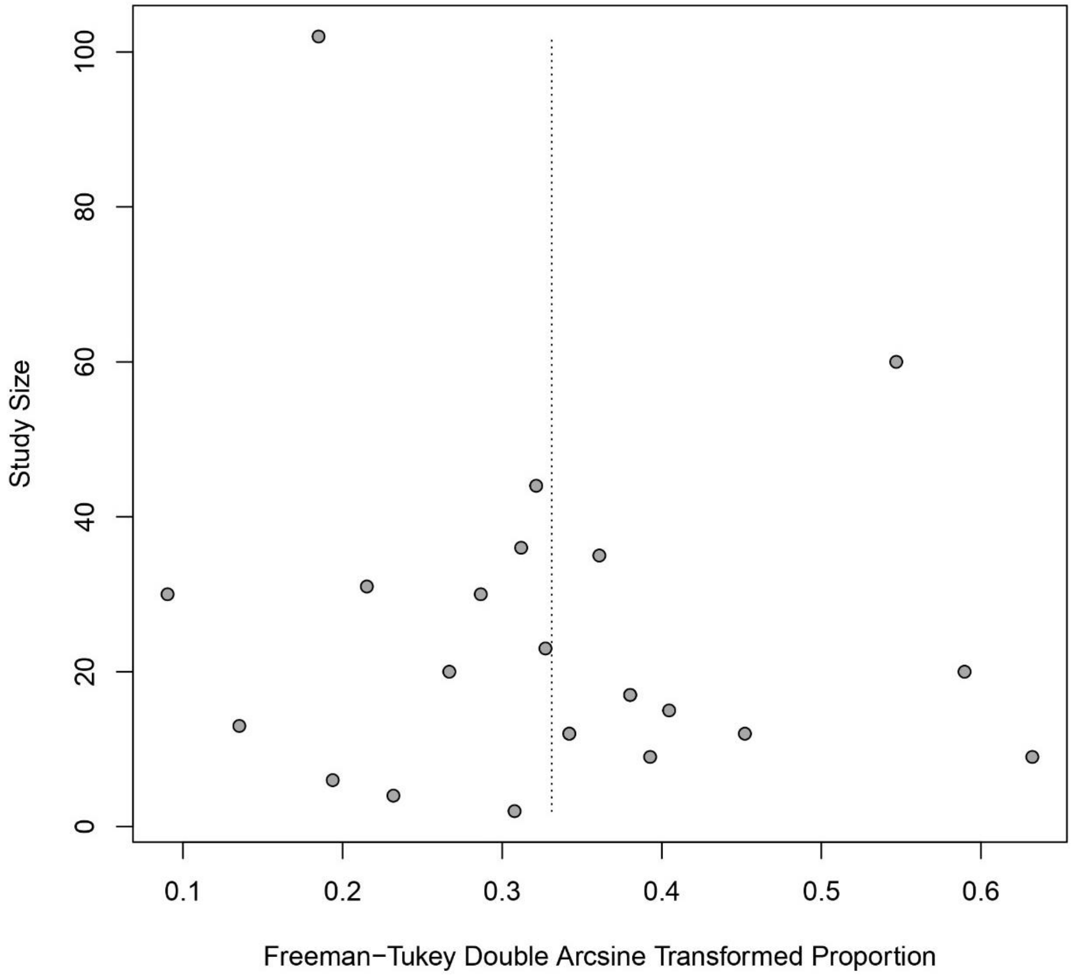

Supplementary Figure 1 – Funnel plot for proportions of overall complications (intra- and postoperative).

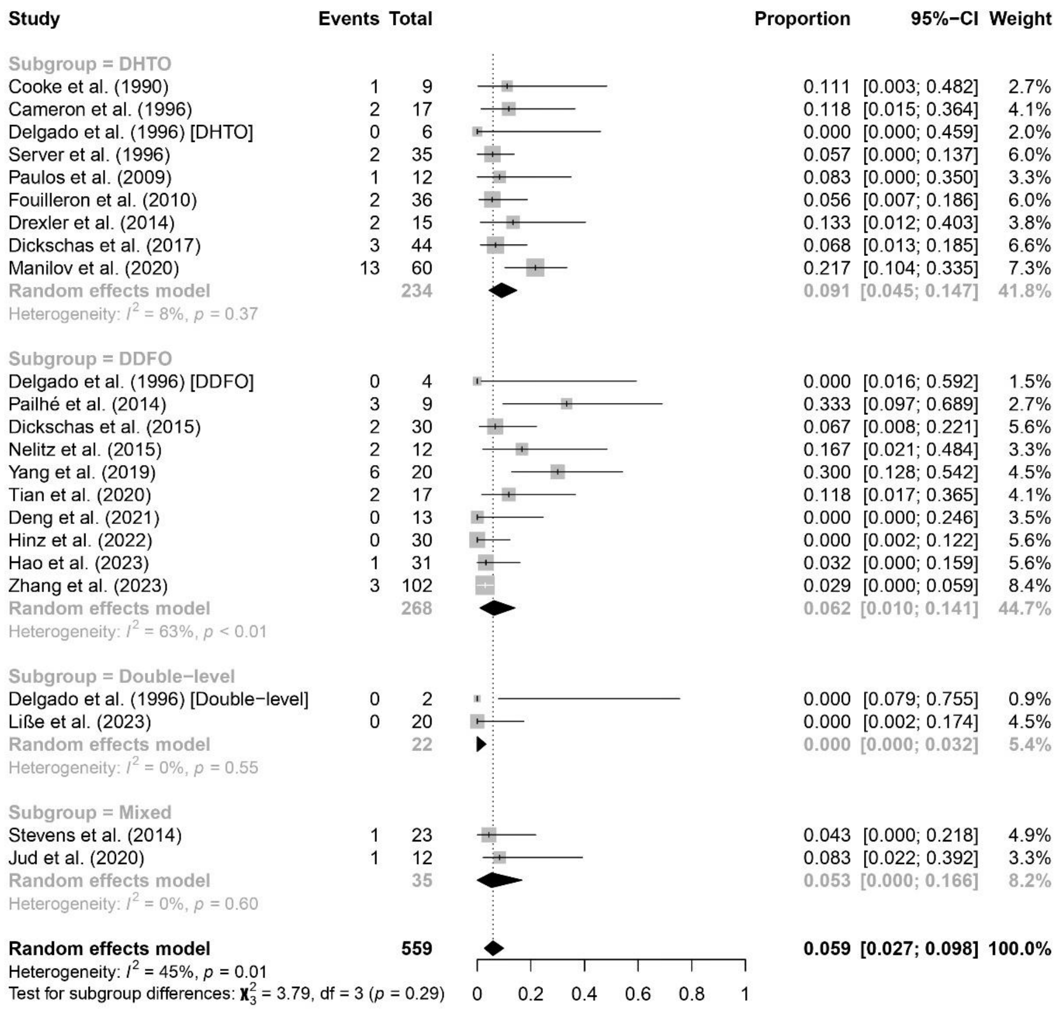

Supplementary Figure 2 – Forest plot for proportions of overall postoperative complications.

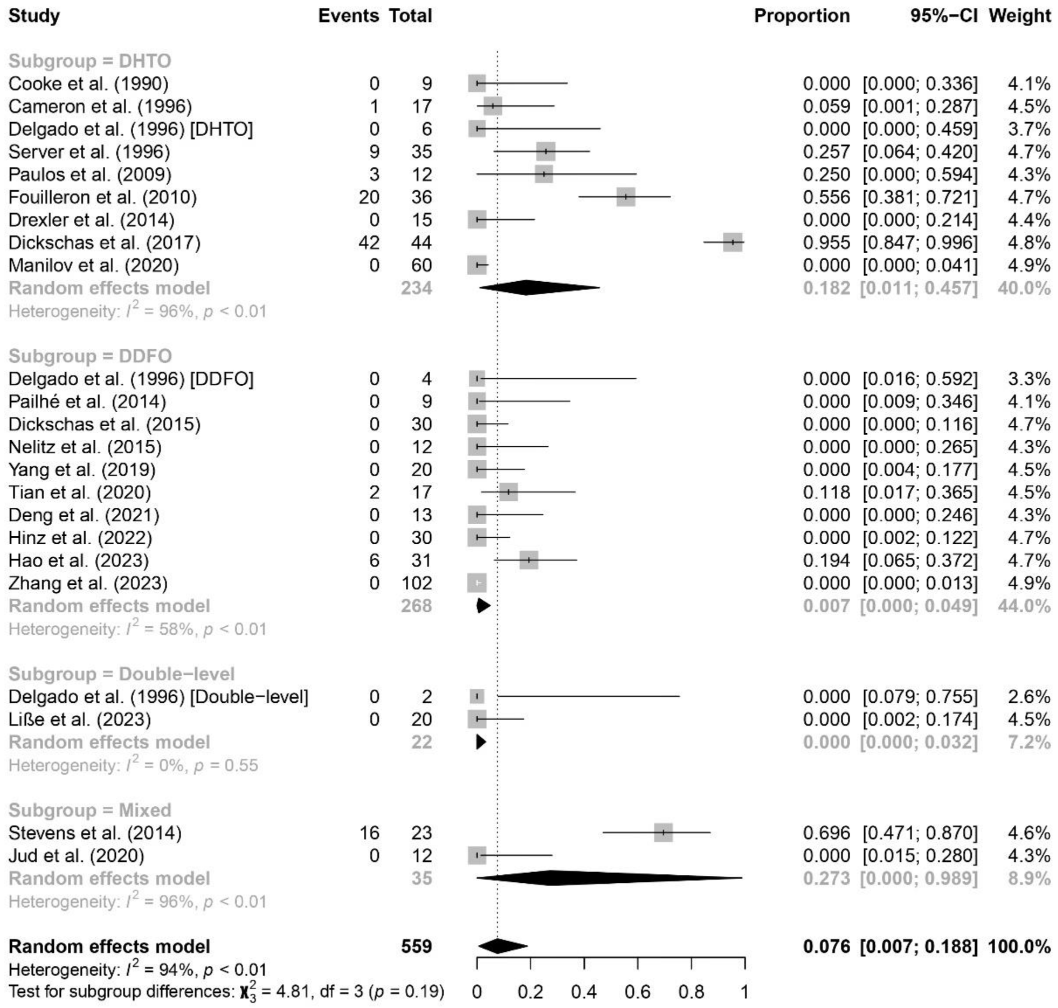

Supplementary Figure 3 – Forest plot for proportions of residual AKP.

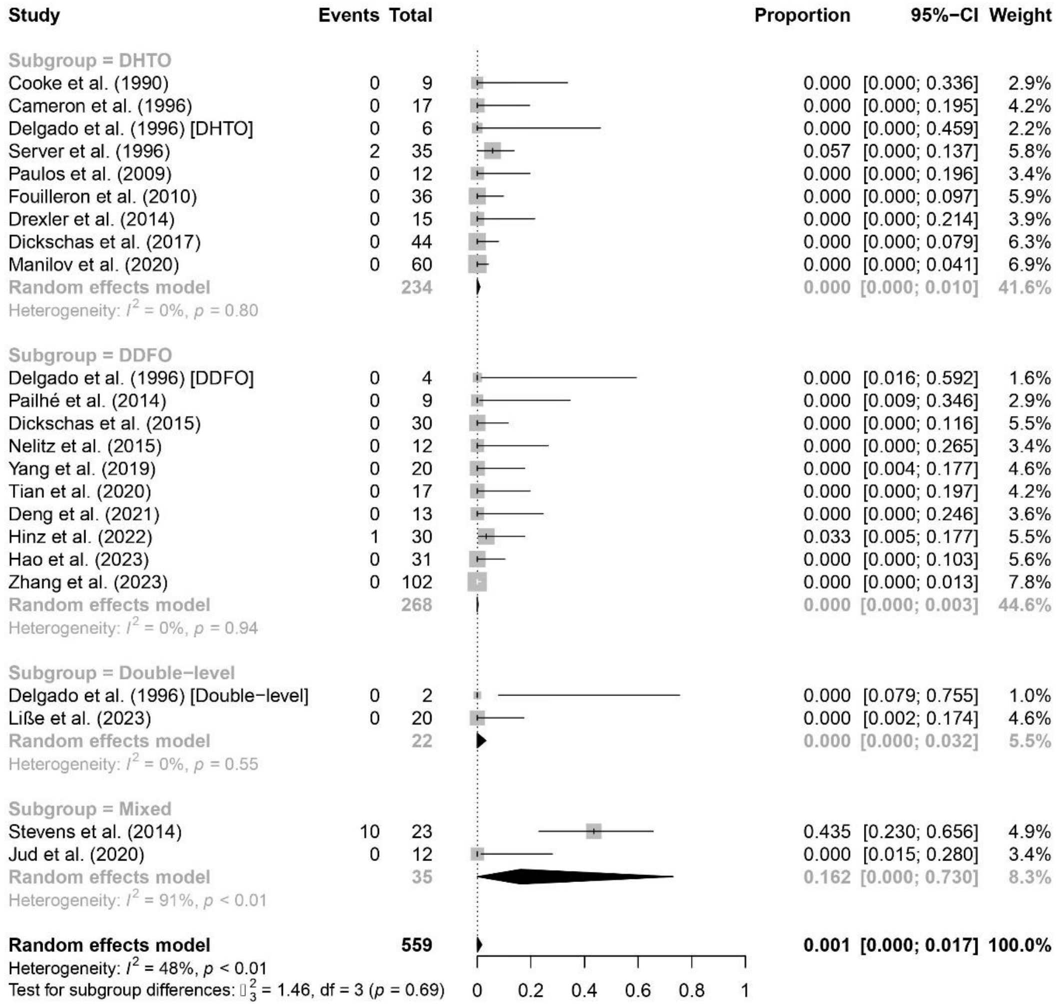

Supplementary Figure 4 – Forest plot for proportions of residual PFI.

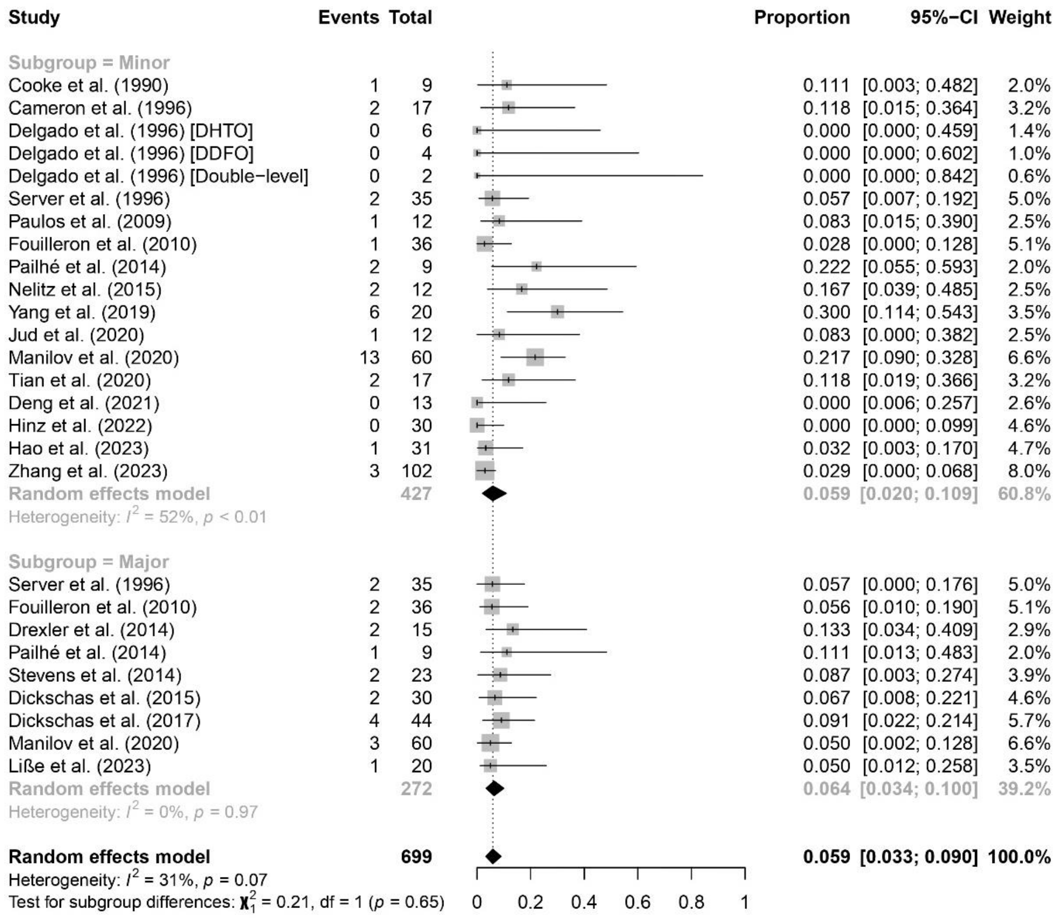

Supplementary Figure 5 – Forest plot for proportions of minor and major complications.

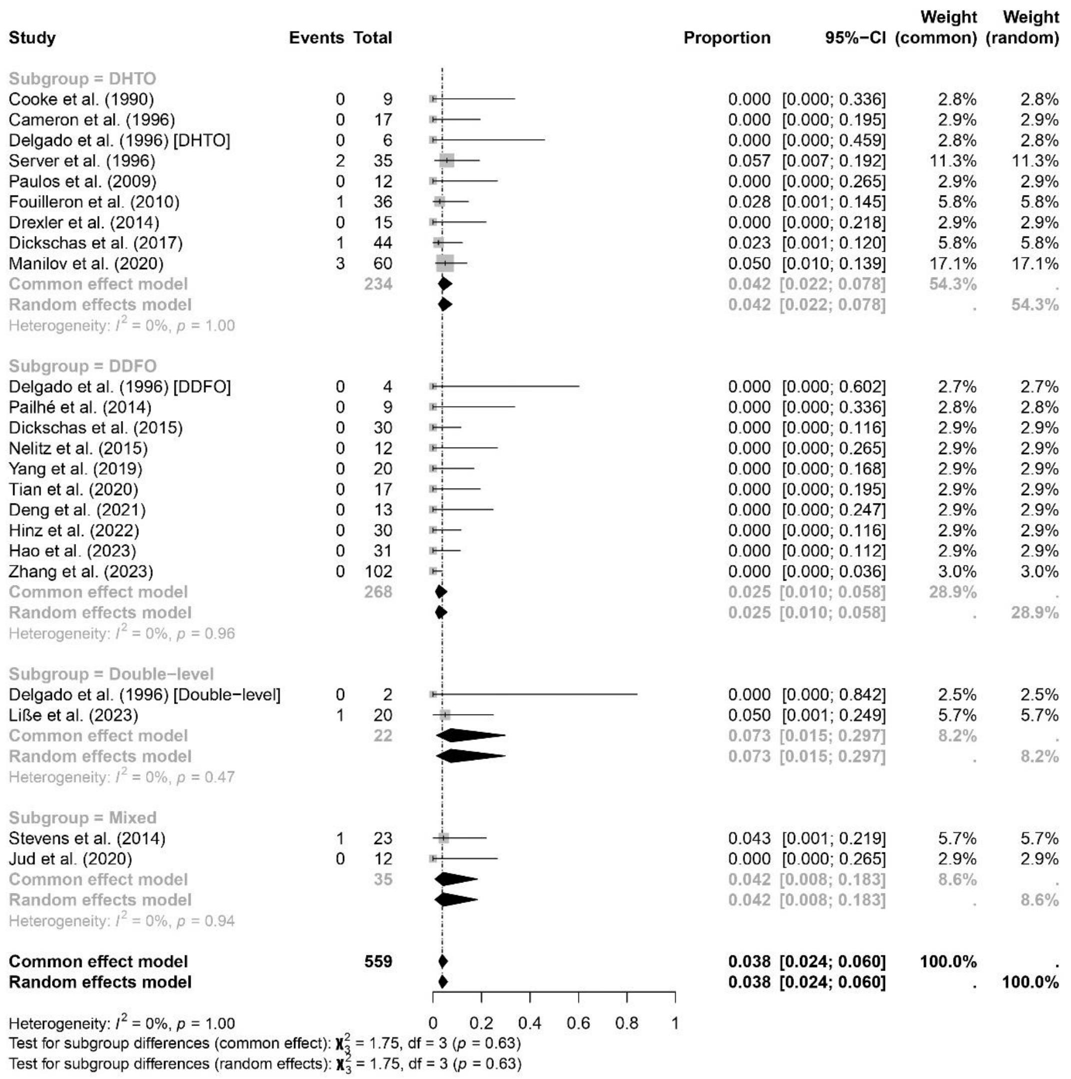

Supplementary Figure 6 – Forest plot for proportions of intraoperative complications.

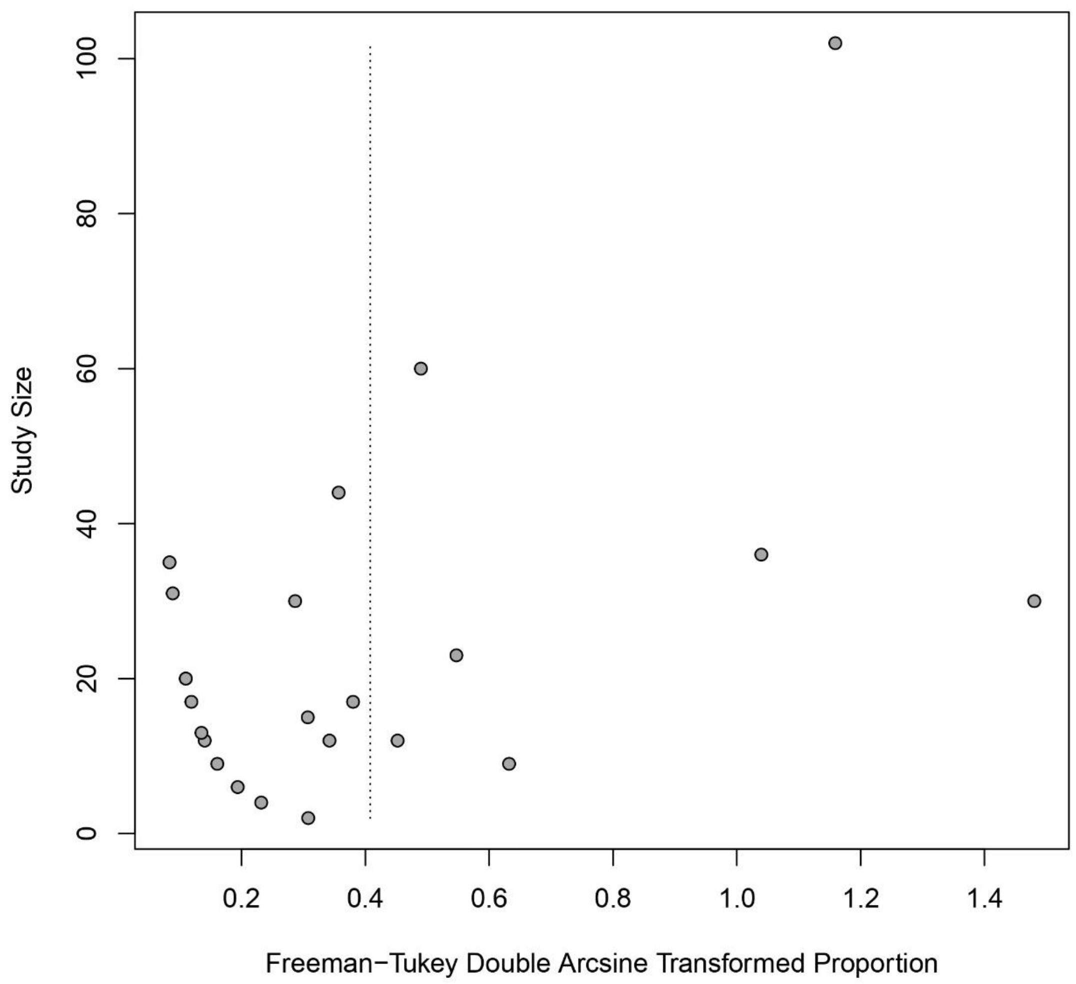

Supplementary Figure 7 – Funnel plot for proportions of reinterventions.

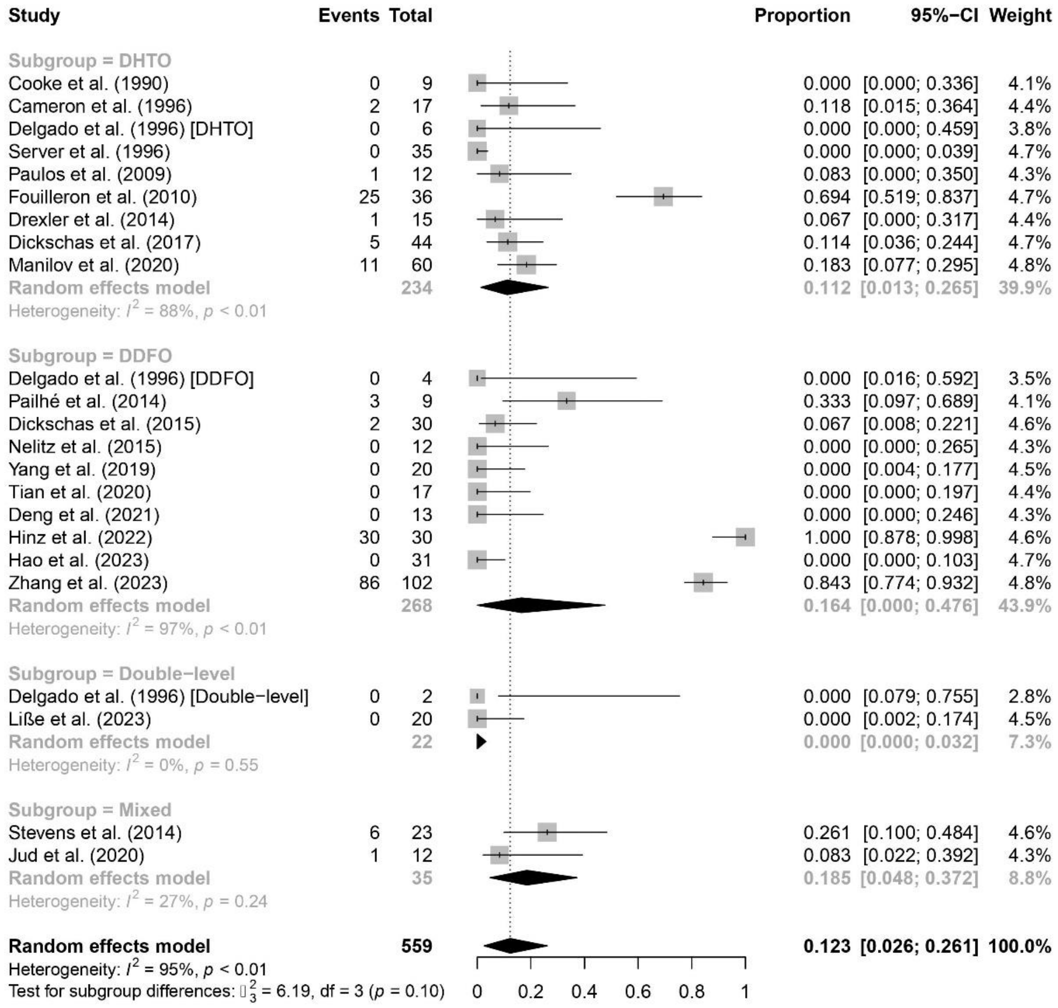

Supplementary Figure 8 – Forest plot for proportions of revision surgeries.

REFERENCES

1. Cooke TD, Price N, Fisher B, Hedden D. The inwardly pointing knee. An unrecognized problem of external rotational malalignment. Clin Orthop Relat Res. 1990(260):56-60.

2. Cameron JC, Saha S. External tibial torsion: an underrecognized cause of recurrent patellar dislocation. Clin Orthop Relat Res. 1996(328):177-84.

3. Delgado ED, Schoenecker PL, Rich MM, Capelli AM. Treatment of severe torsional malalignment syndrome. J Pediatr Orthop. 1996;16(4):484-8.

4. Server F, Miralles RC, Garcia E, Soler JM. Medial rotational tibial osteotomy for patellar instability secondary to lateral tibial torsion. Int Orthop. 1996;20(3):153-8.

5. Paulos L, Swanson SC, Stoddard GJ, Barber-Westin S. Surgical correction of limb malalignment for instability of the patella: a comparison of 2 techniques. Am J Sports Med. 2009;37(7):1288-300.

6. Fouilleron N, Marchetti E, Autissier G, Gougeon F, Migaud H, Girard J. Proximal tibial derotation osteotomy for torsional tibial deformities generating patello-femoral disorders. Orthop Traumatol Surg Res. 2010;96(7):785-92.

7. Drexler M, Dwyer T, Dolkart O, Goldstein Y, Steinberg EL, Chakravertty R, et al. Tibial rotational osteotomy and distal tuberosity transfer for patella subluxation secondary to excessive external tibial torsion: surgical technique and clinical outcome. Knee Surg Sports Traumatol Arthrosc. 2014;22(11):2682-9.

8. Pailhé R, Bedes L, Sales de Gauzy J, Tran R, Cavaignac E, Accadbled F. Derotational femoral osteotomy technique with locking nail fixation for adolescent

femoral antetorsion: surgical technique and preliminary study. J Pediatr Orthop B. 2014;23(6):523-8.

9. Stevens PM, Gililland JM, Anderson LA, Mickelson JB, Nielson J, Klatt JW. Success of torsional correction surgery after failed surgeries for patellofemoral pain and instability. Strategies Trauma Limb Reconstr. 2014;9(1):5-12.

10. Dickschas J, Harrer J, Reuter B, Schwitulla J, Strecker W. Torsional osteotomies of the femur. J Orthop Res. 2015;33(3):318-24.

11. Nelitz M, Dreyhaupt J, Williams SR, Dornacher D. Combined supracondylar femoral derotation osteotomy and patellofemoral ligament reconstruction for recurrent patellar dislocation and severe femoral anteversion syndrome: surgical technique and clinical outcome. Int Orthop. 2015;39(12):2355-62.

12. Dickschas J, Tassika A, Lutter C, Harrer J, Strecker W. Torsional osteotomies of the tibia in patellofemoral dysbalance. Arch Orthop Trauma Surg. 2017;137(2):179-85.

13. Yang GM, Wang YY, Zuo LX, Li FQ, Dai YK, Wang F. Good Outcomes of Combined Femoral Derotation Osteotomy and Medial Retinaculum Plasty in Patients with Recurrent Patellar Dislocation. Orthop Surg. 2019;11(4):578-85.

14. Jud L, Vlachopoulos L, Beeler S, Tondelli T, Fürnstahl P, Fucentese SF. Accuracy of three dimensional-planned patient-specific instrumentation in femoral and tibial rotational osteotomy for patellofemoral instability. Int Orthop. 2020;44(9):1711-7.

15. Manilov R, Chahla J, Maldonado S, Altintas B, Manilov M, Zampogna B. High tibial derotation osteotomy for distal extensor mechanism alignment in patients with squinting patella due to increased external tibial torsion. Knee. 2020;27(6):1931-41.

16. Tian G, Yang G, Zuo L, Li F, Wang F. Femoral derotation osteotomy for recurrent patellar dislocation. Arch Orthop Trauma Surg. 2020;140(12):2077-84.

17. Deng X, Li L, Zhou P, Deng F, Li Y, He Y, et al. Medial patellofemoral ligament reconstruction combined with biplanar supracondylar femoral derotation osteotomy in recurrent patellar dislocation with increased femoral internal torsion and genu valgum: a retrospective pilot study. BMC Musculoskelet Disord. 2021; 22(1):990.

18. Hinz M, Cotic M, Diermeier T, Imhoff FB, Feuerriegel GC, Woertler K, et al. Derotational distal femoral osteotomy for patients with recurrent patellar instability and increased femoral antetorsion improves knee function and adequately treats both torsional and valgus malalignment. Knee Surg Sports Traumatol Arthrosc. 2023;31(8):3091-7.

19. Hao K, Niu Y, Kong L, Wang F. Medial patellofemoral ligament reconstruction combined with derotational distal femoral osteotomy yields better outcomes than isolated procedures in patellar dislocation with increased femoral anteversion. Knee Surg Sports Traumatol Arthrosc. 2023;31(7):2888-96.

20. Liße J, Perl M, Dickschas J. Double-level torsional osteotomy a treatment for the ‘inwardly pointing knee’syndrome. Arch Orthop Trauma Surg. 2023;143(6):2863-75.

21. Zhang ZJ, Di MLQ, Song GY, Li Y, Cao YW, Zheng T, et al. Clinical and Second-look Arthroscopic Results for Derotational Distal Femoral Osteotomy With Medial Patellofemoral Ligament Reconstruction for Recurrent Patellar Dislocation With Increased Femoral Anteversion: A Series of 102 Cases With a Minimum Clinical Follow-up of 2 Years. Am J Sports Med. 2023;51(3):663-71.
